# Supplementary material for: An oncolytic virus as a promising candidate for the treatment of radioresistant oral squamous cell carcinoma
Source: Mol Ther Oncolytics. 2022 Oct 8;27:141–56. doi: 10.1016/j.omto.2022.10.001 (PMC9619351; doi:10.1016/j.omto.2022.10.001)
Supplement: Document S1. Figures S1–S7 and Tables S1 — –S3 and supplementary materials and methods [file mmc1.pdf]

OMTO, Volume *27*

Supplemental information

An oncolytic virus as a promising candidate for the treatment of radioresistant oral squamous cell carcinoma

Shunsuke Gohara, Kosuke Shinohara, Ryoji Yoshida, Ryusho Kariya, Hiroshi Tazawa, Masashi Hashimoto, Junki Inoue, Ryuta Kubo, Hikaru Nakashima, Hidetaka Arita, Sho Kawaguchi, Keisuke Yamana, Yuka Nagao, Asuka Iwamoto, Junki Sakata, Yuichiro Matsuoka, Hisashi Takeshita, Masatoshi Hirayama, Kenta Kawahara, Masashi Nagata, Akiyuki Hirosue, Yoshikazu Kuwahara, Manabu Fukumoto, Seiji Okada, Yasuo Urata, Toshiyoshi Fujiwara, and Hideki Nakayama

Supplementary Materials and Methods

Immunohistochemical staining analysis

Immunohistochemical staining analysis was performed basically as described in main manuscript. We semi-quantified the proportion of Adenovirus-2/5 E1A (E1A)-positive cells among the total number of cancer cells and the staining intensity for E1A. The proportion score of E1A-positive cells was classified as follows: 0, <1%; 1, 1%–10%; 2, 11%–50%; and 3, >50%. The intensity score was classified as follows: 0, lack of intensity; 1, weak, detectable only in high-power fields; 2, moderate, detectable in low-power fields; and 3, strong. The proportion and intensity scores were summed to produce a E1A score ranging 0–6. The HMGB1 and Calreticulin expression was determined by calculating the number of positive cells out of 100 in five random fields (400 × objective). A percentage of positive cells was calculated by dividing the number of positive cells by the total number of cells per sample and multiplying by 100. The antibodies used in above analyses were shown in Supplementary Table S3. In the immunohistochemical staining of tumor tissues obtained from PDX model, the appropriate antibodies shown in Supplementary Table S3 were used.

Histological assessment of vital organs in mice

To evaluate the adverse effects of OBP-301 on vital organs, the heart, liver, and lungs were removed at the end of the treatment experiment. The removed organs were fixed in 10% buffered formalin and H&E stained for histological evaluation.

RT-PCR

Total RNA s isolated using the FastGeneTM RNA Basic Kit (NIPPON Genetics, Tokyo, Japan) and reverse transcribed into cDNA using the ReverTra Ace qPCR RT Kit (Toyobo, Osaka, Japan). PCR was performed using the Thunderbird SYBR qPCR Mix (Toyobo). Data obtained from RT-qPCR were analyzed using the 2−ΔΔCt method, with *glyceraldehyde-3-phosphate dehydrogenase (GAPDH)* as a housekeeping gene. Each sample was run in triplicate. The following primers were used: *E1A* (forward, 5-CCTGAGACGCCCGACATC-3; reverse, 5-GGACCGGAGTCACAGCTATCC-3); *GAPDH* (forward, 5-CAACAGCCTCAAGATCATCAGC-3; reverse, 5-TTCTAGACGGCAGGTCAGGTC-3).

Supplementary Table S1. Antibdy lists

Antigens	Species Antibodies Raised in	Dilution	Supplier, Catalog No.
hTERT	Mouse, monoclonal	1/1000	MBL, #M216-3
CAR	Rabbit, monoclonal	1/1000	Cell Signaling Technology, #16984
STAT3	Rabbit, monoclonal	1/1000	Cell Signaling Technology, #4904
p-STAT3	Rabbit, monoclonal	1/1000	Cell Signaling Technology, #9145
Bcl-xL	Rabbit, monoclonal	1/1000	Cell Signaling Technology, #2764
Cleaved caspase3	Rabbit, monoclonal	1/1000	Cell Signaling Technology, #9664
LC3 I/II	Rabbit, monoclonal	1/1000	Cell Signaling Technology, #12741
p62	Rabbit, polyclonal	1/1000	MBL, #PM045
β-actin	Mouse, monoclonal	1/2000	Sigma-Aldrich, A5316

Supplementary Table S2. Primer sets for RT-qPCR

Gene	sequence 5'-3' Forward	sequence 5'-3' Reverse
hTERT	TTCCTGCACTGGCTGATGAGTGT	CGCTCGGCCCTCTTTTCTCTG
CAR	AGCCTTCAGGTGCGAGATGTTACG	TACGACAGCAAAAGATGATAAGAC
GAPDH	GGGAAGGTGAAGGTCGGAGTC	AGCAGAGGGGGCAGAGATGAT

Supplementary Table S3. Antibdy lists

Antigens	Species Antibodies Raised in	Dilution	Supplier, Catalog No.
hTERT	Mouse, monoclonal	1/100	Wako, #TMab-6
p-STAT3	Rabbit, monoclonal	1/100	Cell Signaling Technology, #9145
Bcl-xL	Rabbit, monoclonal	1/200	Cell Signaling Technology, #2764
Anti-Coxsackie Adenovirus Receptor/hCAR	Rabbit, polyclonal	1/100	Abcam, ab133380
Adenovirus-2/5 E1A	Rabbit, polyclonal	1/100	Santa Cruz Biothechnology, M73
p62	Rabbit, polyclonal	1/200	Proteintech, 18420-0-AP
HMGB1	Rabbit, polyclonal	1/100	Proteintech, 10829-1-AP
Calreticulin	Rabbit, polyclonal	1/200	Proteintech, 27298-1-AP

A

RT-PCR

CAR *hTERT*

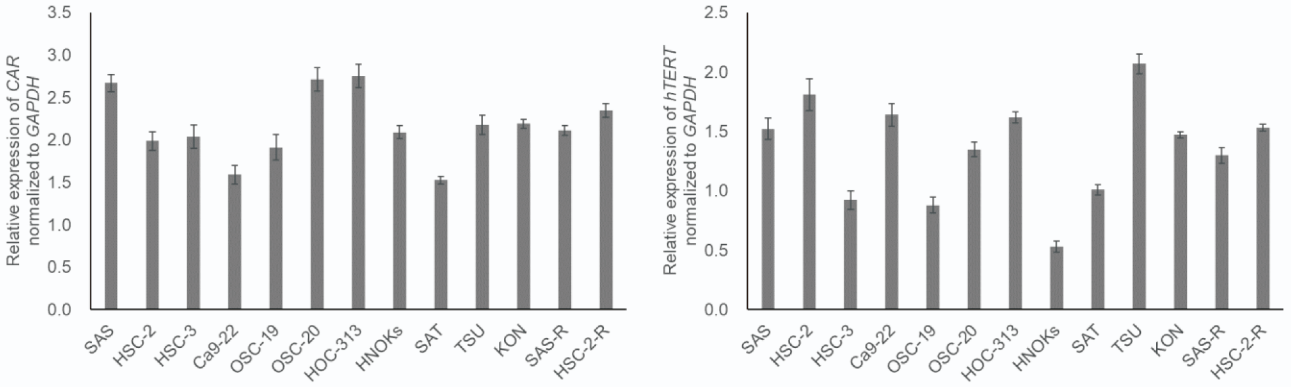

B

Western blotting

CAR hTERT

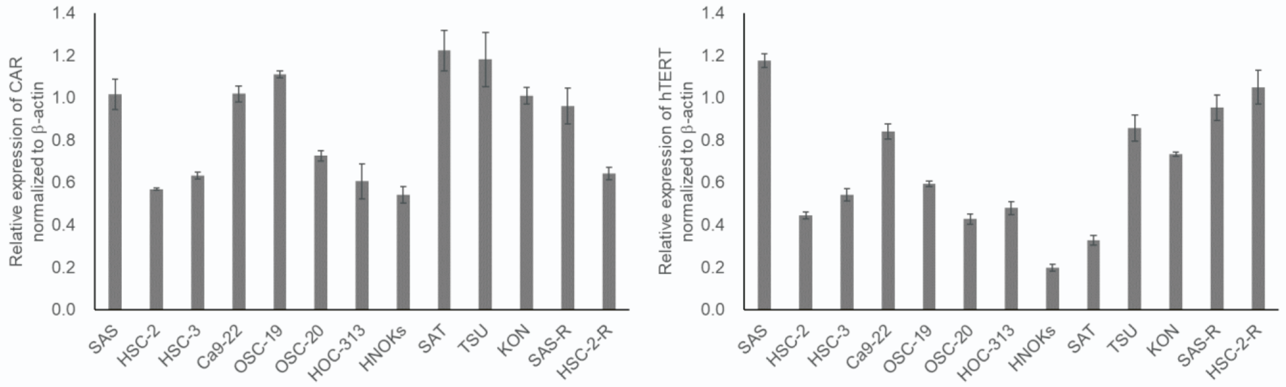

Figure S1. Results of semi-quantitative analysis of RT-PCR and western blotting.

A) The graph of semi-quantitative analysis of RT-PCR.. The result of CAR (lest panel). The result of hTERT (right panel). The results are shown as the means ± s.d. of three independent experiments.

B) The graph of semi-quantitative analysis of western blotting. These results are shown as the means of at least three independent experiments performed in triplicate. The result of CAR (lest panel). The result of hTERT (right panel). The results are shown as the means ± s.d. of three independent experiments.

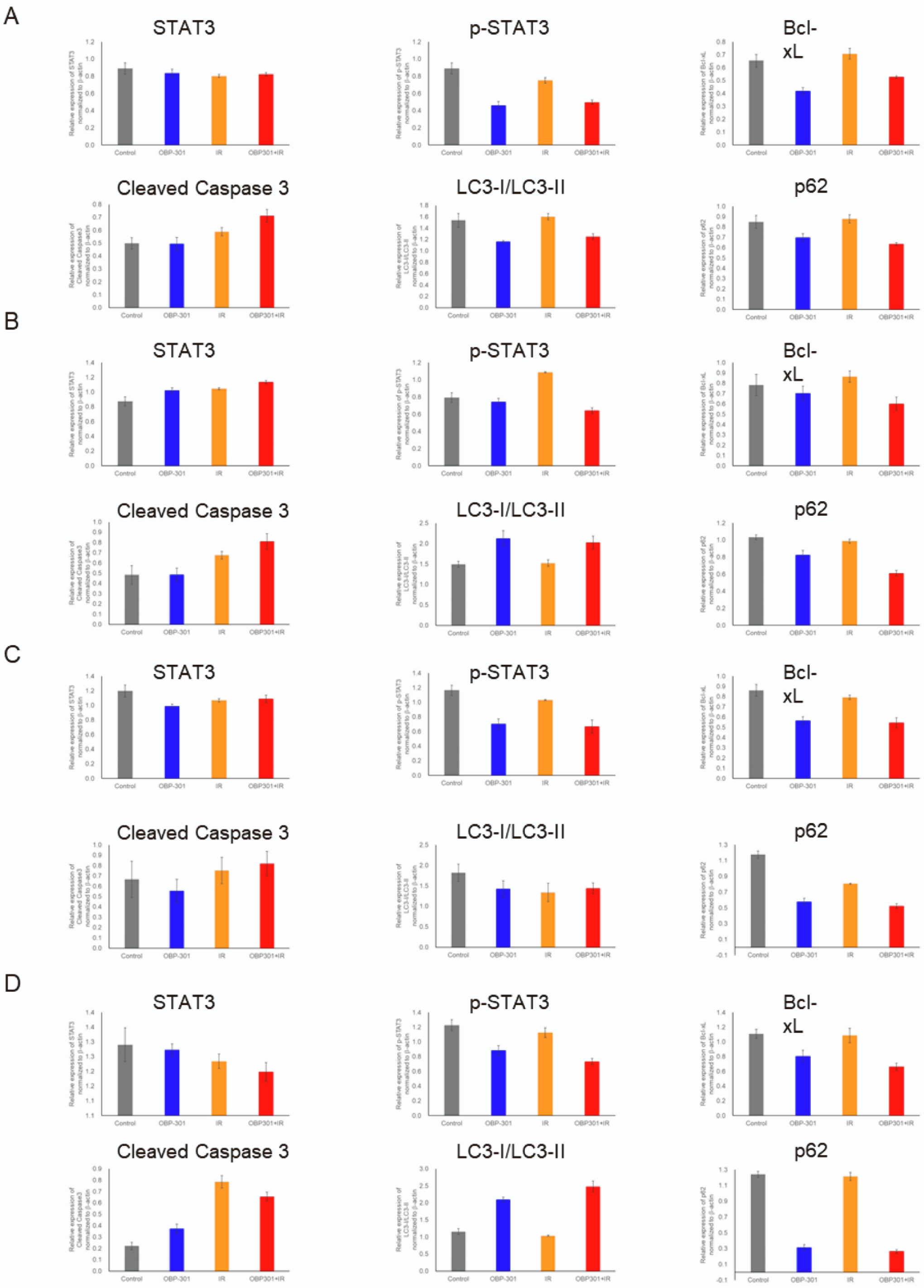

Figure S2. Results of semi-quantitative analysis of western blotting.

A) The graph of semi-quantitative analysis of western blotting in SAS. The results are shown as the means ± s.d. of three independent experiments.

B) The graph of semi-quantitative analysis of western blotting in SAS-R. The results are shown as the means ± s.d. of three independent experiments.

C) The graph of semi-quantitative analysis of western blotting in HSC-2. The results are shown as the means ± s.d. of three independent experiments.

D) The graph of semi-quantitative analysis of western blotting in HSC-2-R. The results are shown as the means ± s.d. of three independent experiments.

A

Age	83
Sex	Female
Primary site	Right of the tongue
cTNM	cT3N0M0
Pattern of invasion	WPOI-5
Initial Treatment	Extensive surgery
Prognosis	Dead from cancer

B

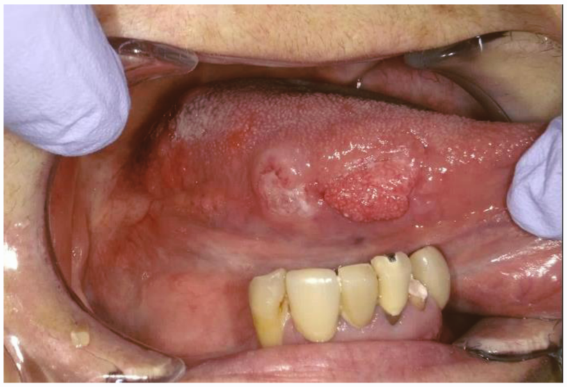

C

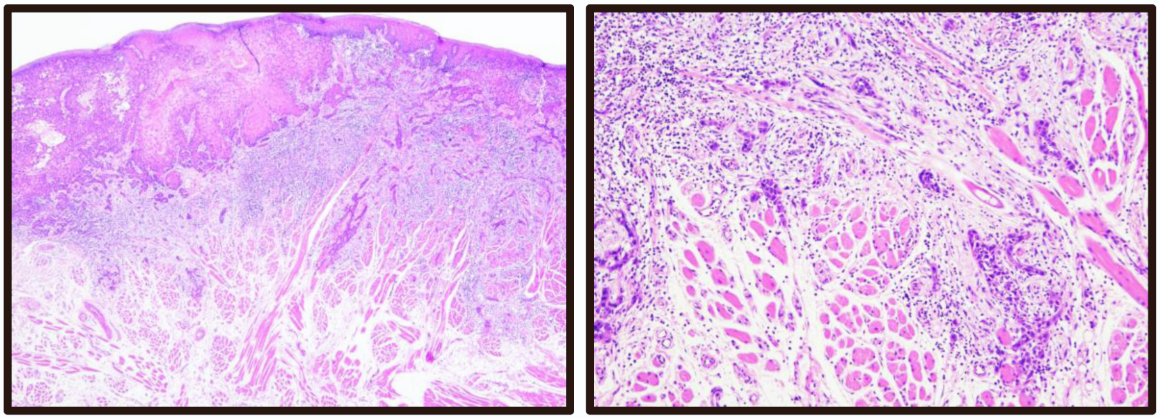

D

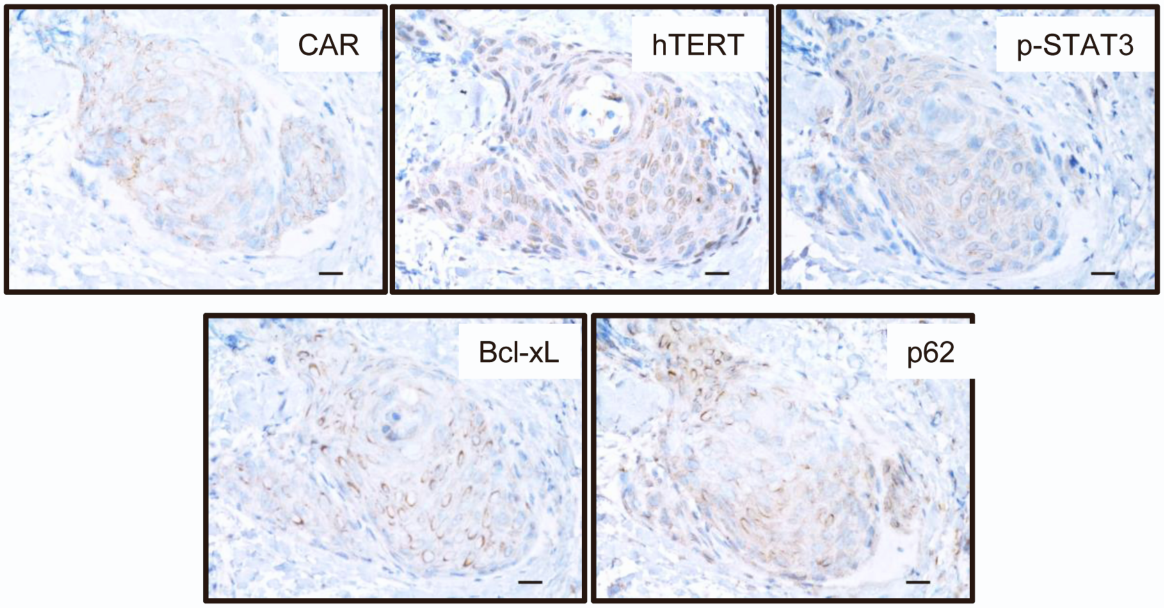

Figure S3. Patient information used to establish the PDX model.

A) Detailed clinical information of the patient.

B) Intraoral photographs of the patient.

C) Representative hematoxylin and eosin staining photomicrographs of the resected tumor (Right; High-power field, Left; Low-power field).

D) The results of immunohistochemical staining of tumors in PDX model under no treatment condition.

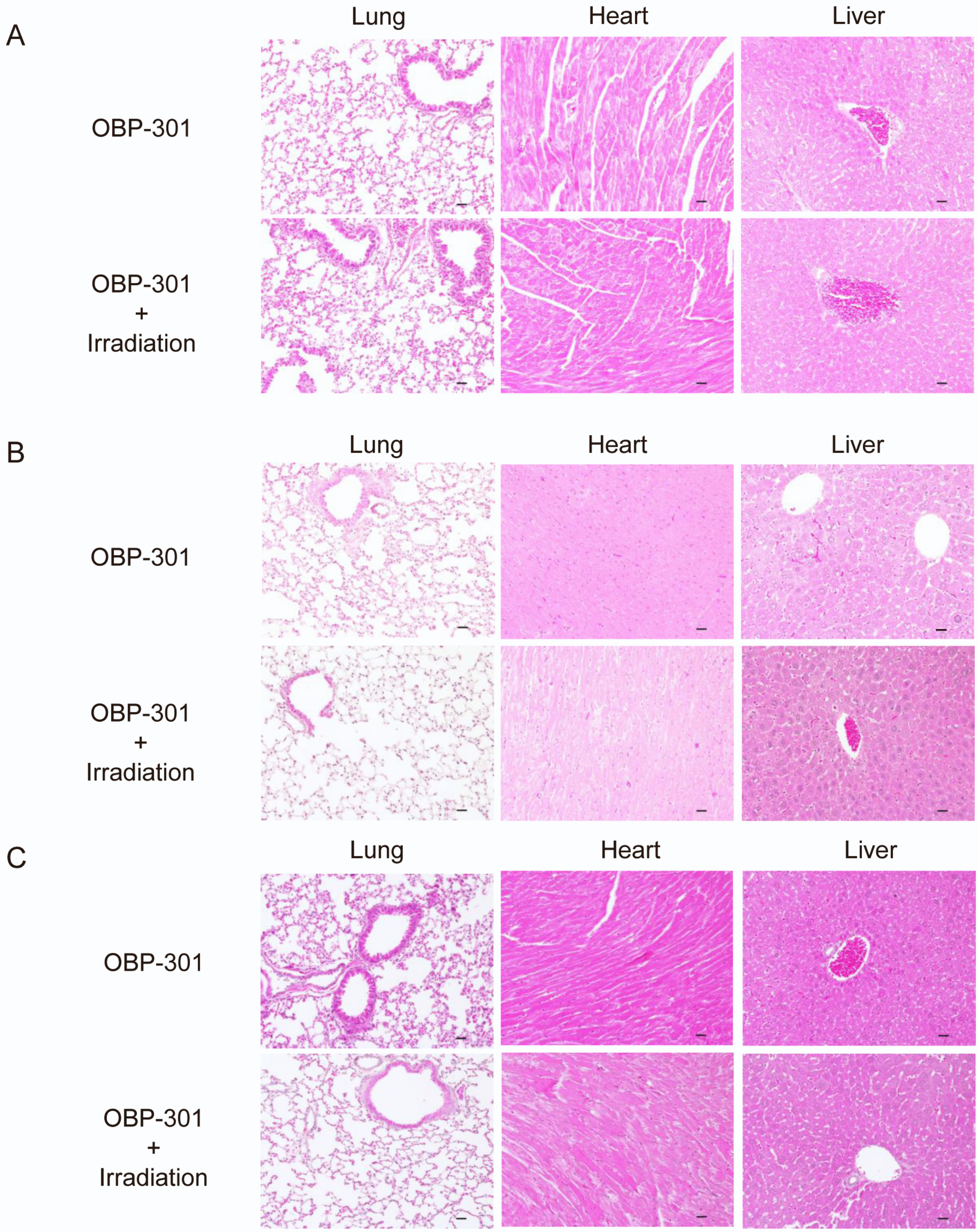

Figure S4. Histology of vital organs in a mouse subcutaneous transplantation model with treatment experiments.

A) Representative hematoxylin and eosin staining of vital organs in a mouse model of subcutaneously implanted SAS. Original magnification, ×200. Scale bar: 20 μm.

B) Representative hematoxylin and eosin staining of vital organs in a mouse model of subcutaneously implanted SAS-R. Original magnification, ×200. Scale bar: 20 μm.

C) Representative hematoxylin and eosin staining of vital organs in a PDX model. Original magnification, ×200. Scale bar: 20 μm.

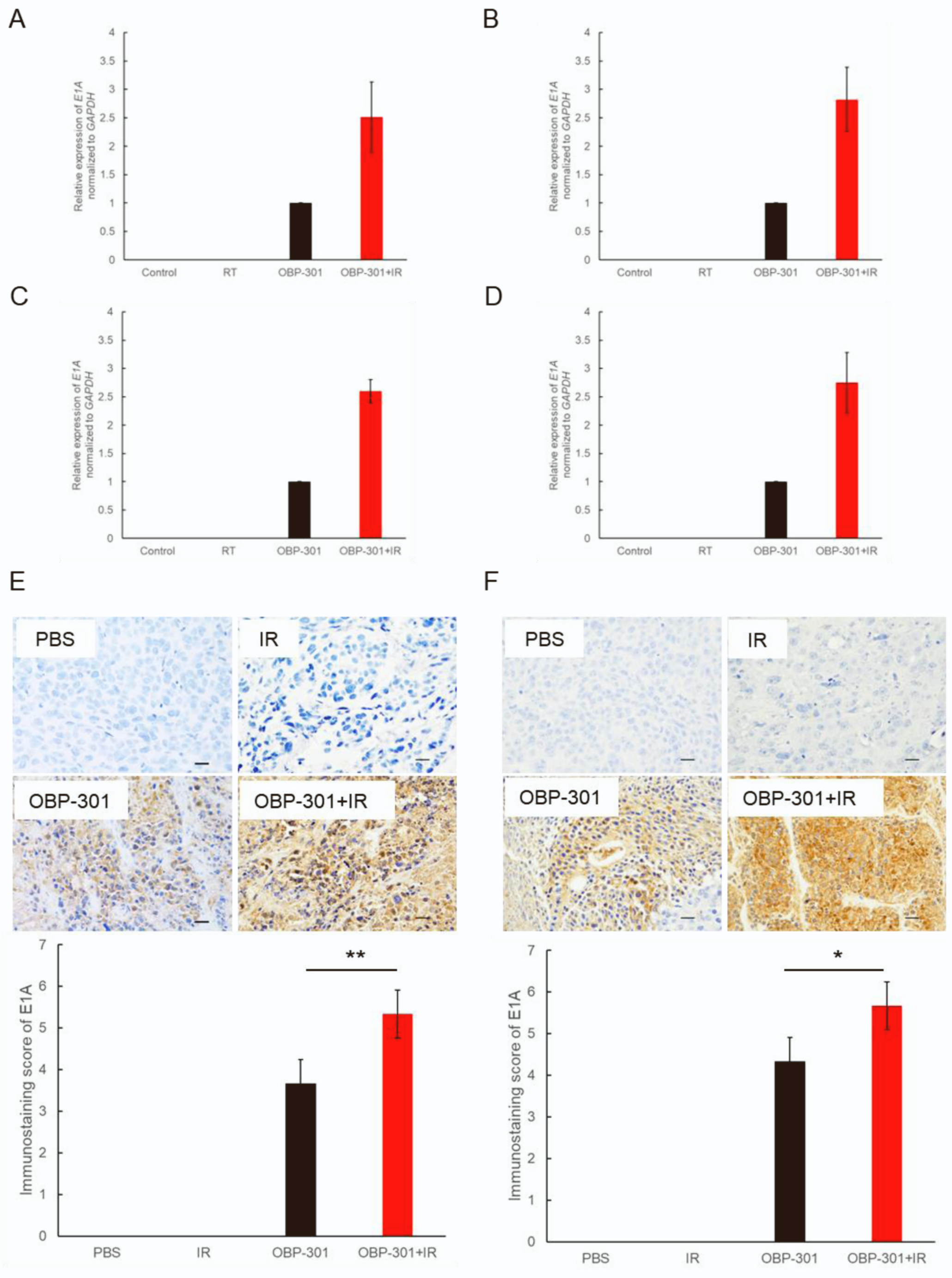

Figure S5. Analysis of the effect of irradiation on the infection efficiency of OBP-301.
Representative immunohistochemical stainings of E1A in cell-derived xenograft model by using SAS (upper). NT, no treatment; RT, radiotherapy. Original magnification, ×400. Scale bar=20 mm. The immunostaining score obtained from three independent experiments were calculated and statistically analyzed. The results are shown as the means ± s.d. of three independent experiments (bottom). *p < 0.05.
Representative immunohistochemical stainings of E1A in cell-derived xenograft model by using SAS-R (upper). NT, no treatment; RT, radiotherapy. Original magnification, ×400. Scale bar =20 mm. The immunostaining score obtained from three independent experiments were calculated and statistically analyzed. The results are shown as the means ± s.d. of three independent experiments (bottom). **p < 0.01.

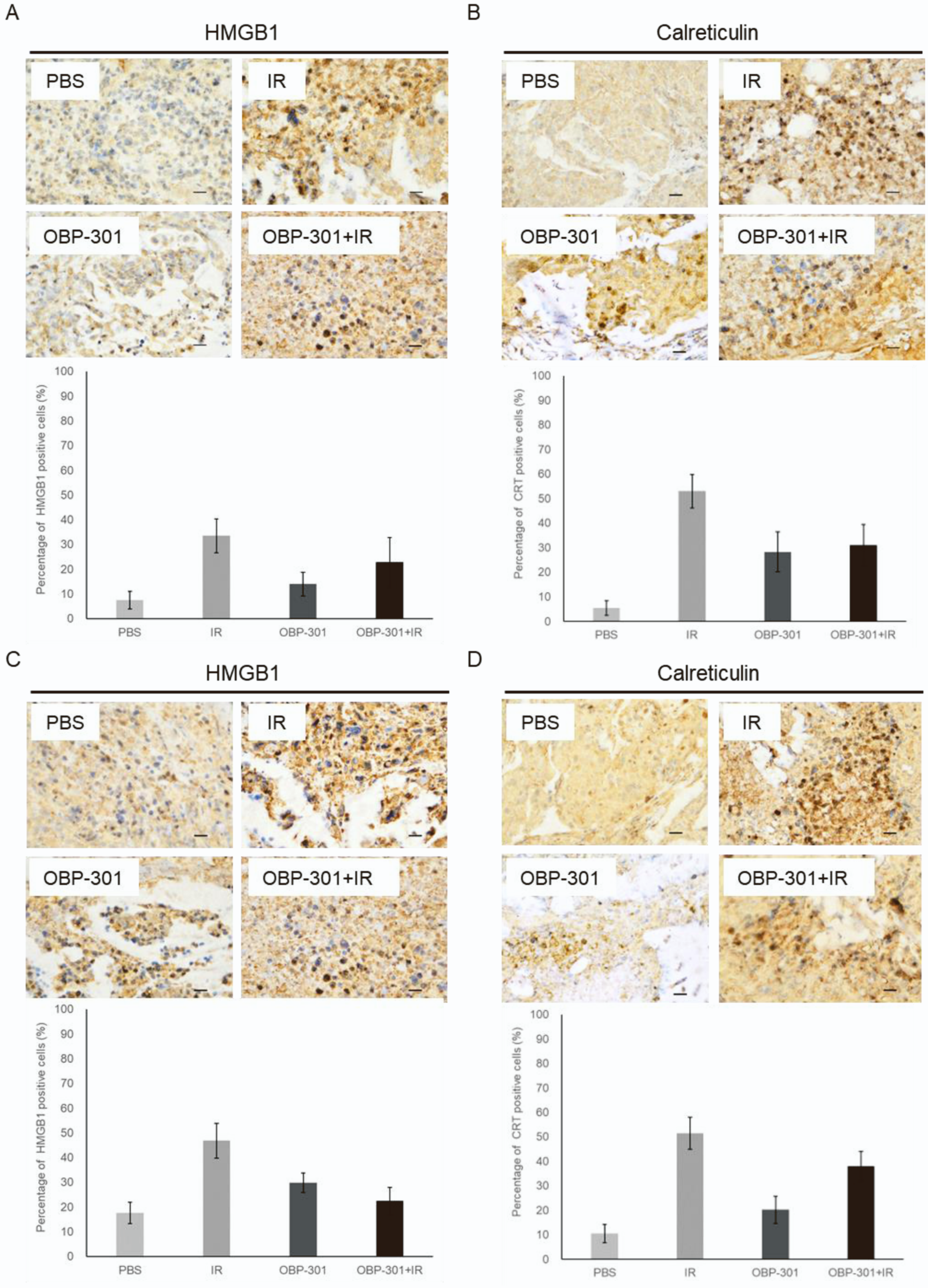

Figure S6. Immunohistochemical staining analysis of HMGB1 and calreticulin *in vivo*.
(A-D) Representative images of immunostaining of HMGB1 and Calreticulin using samples obtained from the CDX model after the experiment (upper). Scale bar: 20 μm. The number of positive cells obtained from three independent experiments were calculated and statistically analyzed. The results are shown as the means ± s.d. of three independent experiments (bottom).

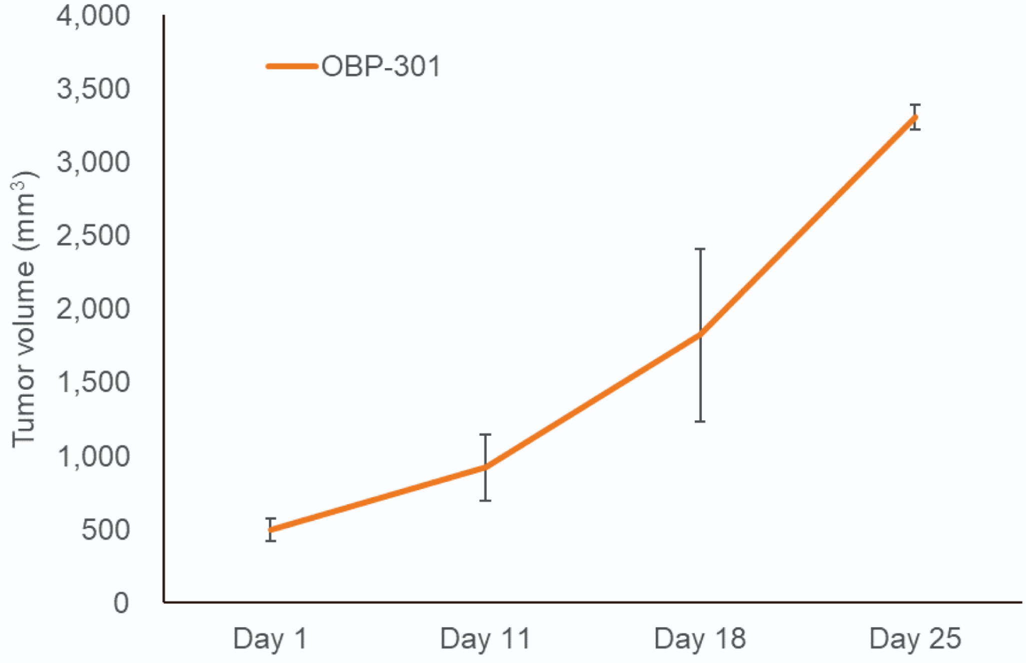

Figure S7. Preliminary experiments with OBP-301 monotherapy in PDX model.
The graphs of tumor volume transition of OBP-301 monotherapy in PDX model. Mean ± S.D. of three independent experiments was calculated. n=3 per group.
