# Supplementary material for: An oncolytic virus as a promising candidate for the treatment of radioresistant oral squamous cell carcinoma
Source: Mol Ther Oncolytics. 2022 Oct 8;27:141–56. doi: 10.1016/j.omto.2022.10.001 (PMC9619351; doi:10.1016/j.omto.2022.10.001)
Supplement: Document S2. Article plus supplemental information [file mmc2.pdf]

Molecular Therapy
Oncolytics
Original Article

An oncolytic virus as a promising candidate for the treatment of radioresistant oral squamous cell carcinoma

Shunsuke Gohara,[1,7] Kosuke Shinohara,[1,7] Ryoji Yoshida,[1] Ryusho Kariya,[6] Hiroshi Tazawa,[2] Masashi Hashimoto,[2] Junki Inoue,[1] Ryuta Kubo,[1] Hikaru Nakashima,[1] Hidetaka Arita,[1] Sho Kawaguchi,[1] Keisuke Yamana,[1] Yuka Nagao,[1] Asuka Iwamoto,[1] Junki Sakata,[1] Yuichiro Matsuoka,[1] Hisashi Takeshita,[1] Masatoshi Hirayama,[1] Kenta Kawahara,[1] Masashi Nagata,[1] Akiyuki Hirosue,[1] Yoshikazu Kuwahara,[3] Manabu Fukumoto,[4] Seiji Okada,[5] Yasuo Urata,[5] Toshiyoshi Fujiwara,[2] and Hideki Nakayama[1]

[1]Department of Oral and Maxillofacial Surgery, Faculty of Life Sciences, Kumamoto University, Kumamoto, Japan; [2]Department of Gastroenterological Surgery, Graduate School of Medicine, Dentistry and Pharmaceutical Sciences, Okayama University, Okayama, Japan; [3]Radiation Biology and Medicine, Faculty of Medicine, Tohoku Medical and Pharmaceutical University, Sendai, Japan; [4]Pathology Informatics Team, RIKEN Center for Advanced Intelligence Project, Chuo-ku, Tokyo, Japan; [5]Division of Hematopoiesis, Joint Research Center for Human Retrovirus Infection and Graduate School of Medical Sciences, Kumamoto University, Kumamoto, Japan; [6]Oncolys Biopharma, Inc., Tokyo, Japan

We evaluated the usefulness of an oncolytic virus (Suratadenoturev; OBP-301) against radioresistant oral squamous cell carcinoma. We confirmed the expression of human telomerase reverse transcriptase and the coxsackievirus and adenovirus receptor in cell lines. Also, we examined the potential presence in a patient who has received existing therapy that is amenable to treatment with OBP-301. We evaluated: (1) the antitumor effects of OBP-301 alone and in combination with radiotherapy on radioresistant cell lines, (2) the molecular mechanism underlying the radiosensitizing effect and cell death increased by the combination therapy, and (3) the antitumor effect of the combination therapy *in vivo* using xenograft models (a radioresistant cell line-derived xenograft in mouse and a patient-derived xenograft). Human telomerase reverse transcriptase and the coxsackievirus and adenovirus receptor were expressed in all cell lines. OBP-301 decreased the proliferative activity of these cell lines in a concentration-dependent manner, and significantly enhanced the antitumor effect of irradiation. Phosphorylated STAT3 and its downstream molecules, which correlated with apoptosis and autophagy, showed significant changes in expression after treatment with OBP-301. The combination therapy exerted a significant antitumor effect versus radiotherapy alone in both xenograft models. Combination of OBP-301 with radiotherapy exerts a synergistic effect and may represent a promising treatment for radioresistant oral squamous cell carcinoma.

INTRODUCTION

Oral squamous cell carcinoma (OSCC) is one of the most common types of cancer of the oral cavity. However, the survival rate has not improved despite advancements in diagnostic modalities and treatments.[1] Thus, the prognosis of advanced OSCC remains poor, with a 5-year survival rate of approximately 50%.[2] This stagnation in the survival rate is mainly attributed to the existence of high-grade malignant cells that display important hallmarks of cancer, such as resistance to chemotherapy or radiotherapy, abnormal proliferation, and invasion or metastasis.[3] Among them, radioresistance is a serious problem that prevents improvement in treatment outcomes of radiotherapy, an important treatment option in OSCC.[4] Recently, we established clinically relevant radioresistant (CRR) cell lines by irradiating cells with >60 Gy for 5 weeks at 2 Gy per day, as in actual clinical practice.[5] Based on preclinical research that used the CRR cell lines to investigate the molecular mechanism involved in radioresistance,[6] these cell lines are regarded as a good experimental resource.

In Japan, 60% of patients age ≥70 years are newly diagnosed with cancer.[7] According to the guidelines established by the National Center for Biotechnology Information, the standard therapy for OSCC is composed of radical therapy with extensive resection of tumors and postoperative concurrent chemoradiotherapy (CRT).[8] However, since old age is associated with a poorer reserve force, the application of this regimen in elderly patients with advanced OSCC is difficult. Of note, many of these patients are undergoing radiotherapy. Therefore, there is an urgent need for new approaches to overcome treatment resistance and to provide new treatment options for patients with OSCC.

Received 24 March 2022; accepted 4 October 2022;
https://doi.org/10.1016/j.omto.2022.10.001.

[7]These authors contributed equally

Correspondence: Ryoji Yoshida, Department of Oral and Maxillofacial Surgery, Graduate School of Life Sciences, Kumamoto University, Honjo 1-1-1, Chuo-ku, Kumamoto 860-8556, Japan.
E-mail: ryoshida@kumamoto-u.ac.jp

© 2022 The Author(s).
This is an open access article under the CC BY license (http://creativecommons.org/licenses/by/4.0/).

OBP-301 is a telomerase-specific tumor-lysing adenovirus developed by Fujiwara et al.[9] It infects target cells via its receptor, coxsackievirus and adenovirus receptor (CAR), whose expression correlates with the infection efficacy of the adenovirus.[10] Following incorporation into the host DNA, OBP-301 produces adenoviral E1A and E1B in response to the promoter activity of human telomerase reverse transcriptase (hTERT). Its activity is related to that of telomeres, which are structural proteins at the ends of chromosomes that protect the DNA.[10,11] Ultimately, OBP-301 causes cell death at relatively high rates in hTERT-positive cancer cells in an hTERT-expression-dependent manner. In contrast, the replication and cytotoxicity of the virus are significantly limited in normal somatic cells.[9,12]

Preclinical research has demonstrated the antitumor effects of OBP-301 in numerous malignancies.[9,12–14] In phase I clinical trials in the United States, OBP-301 has shown an efficacious and safe profile against several types of solid tumors.[15] Furthermore, it has been reported that monotherapy and radiation therapy can be used in combination to further improve the antitumor effect.[16] In head and neck squamous cell carcinoma (HNSCC), including OSCC, several studies evaluated mainly the antitumor effects of single-agent administration.[17–19] Some studies reported the combined effects of treatment with anticancer drugs and radiation *in vitro* and *in vivo*.[17,20–22] However, there are no reports examining the antitumor effect or mechanism underlying the development of resistance using radioresistant OSCC cell lines and a patient-derived xenograft (PDX) model.

It is thought that radiation exerts its therapeutic effects on cancer cells by inducing various types of cell death (i.e., apoptosis, mitotic catastrophe, necrosis, and autophagy) and by inhibiting cell proliferation.[23] Recently, our group reported that radiation-induced regulatory cell death can be classified into three categories, namely apoptosis, autophagy-dependent cell death, and necrosis.[24] OBP-301 possesses strong antitumor activity that can lyse cancer cells by specifically proliferating in them.[9] Moreover, when OBP-301 is used in combination with existing treatment modalities, it can enhance the antitumor effects through various mechanisms, such as the DNA repair machinery, enhancing apoptosis, and local immune modification of tumors.[22,25,26] Several studies have identified radioresistance-related molecules using radioresistant OSCC cell lines. Reports in the past have focused on molecules identified by gene expression analyses to elucidate radioresistance mechanisms.[27–29] However, few studies have been reported in OSCC with respect to mechanisms of radioresistance, particularly using multiple CRR cells established by the routine clinical irradiation of OSCC cell lines.

In this study, we investigated *in vitro* and *in vivo* whether the combination of OBP-301 radiotherapy can overcome radioresistance in OSCC using a useful research model, namely CRR cells. We also investigated the mechanism of cell death induced by this combination in OSCC. In addition, we further validated the usefulness of this treatment *in vivo*, using a CRR cell-line-derived xenograft (CRR-CDX) model and the PDX model as a useful preclinical model.

RESULTS

OBP-301 target molecules are expressed in OSCC cell lines

We analyzed the expression of CAR and hTERT, which have been reported as therapeutic target molecules of OBP-301,[30] in OSCC cell lines and human normal oral keratinocytes (HNOKs) using real-time PCR and western blotting. Expression of *CAR* mRNA was detected in all cell lines, including HNOK (Figure 1A). Expression of *hTERT* mRNA was confirmed in all OSCC cell lines, but not in HNOK (Figure 1A). In the western blotting analysis, although there was a difference in expression, CAR protein was detected in all cell lines (including HNOK). Expression of hTERT protein was detected in all OSCC cell lines, but not in HNOK (Figure 1B).

hTERT expression correlated with response to CRT and prognosis in patients with advanced OSCC

To elucidate the clinical significance of hTERT in OSCC, we performed expression analysis of hTERT by immunohistochemical staining. In addition, we investigated the clinicopathological significance of hTERT in preoperative specimens obtained from 50 patients with advanced OSCC who underwent preoperative CRT. As shown in the representative images of immunohistochemical staining (Figure 1C), various hTERT expression patterns were confirmed in clinical OSCC. In the clinicopathological analysis, the high expression status of hTERT was significantly correlated with the clinical stage ($p = 0.001$), mode of invasion ($p = 0.015$), loco-regional recurrence ($p = 0.023$), and poor pathological response to CRT ($p = 0.016$) (Table 1). Moreover, 5-year disease-free survival (DFS) rates for patients with high hTERT expression were significantly lower than those for patients with low hTERT expression ($p = 0.018$) (Figure 1D, right). Furthermore, after adjusting for various clinicopathological factors, the influence of hTERT expression on DFS (hazard ratio, 3.241; 95% CI 1.112–9.992; $p = 0.031$) (Table 2) remained in the Cox proportional hazards regression model. On the other hand, although the 5-year overall survival (OS) rate tended to be lower in patients with low hTERT expression, the difference was not statistically significant ($p = 0.420$) (Figure 1D, left).

In vitro radiosensitizing effect of OBP-301 on CRR OSCC cell lines

To investigate whether OBP-301 contributed to the treatment of radiation-resistant OSCC, we analyzed the antitumor and radiosensitizing effects of OBP-301 on OSCC cells, including CRR cell lines. We first confirmed the antitumor effect of OBP-301 alone in OSCC cells. As shown in Figure 2A, treatment with OBP-301 exerted a concentration-dependent antitumor effect on OSCC cell lines (Figure 2A). Next, we explored the radiosensitizing effect of OBP-301 on OSCC cell lines, including CRR cells. The results of the modified high-density survival (MHDS) assay showed that OBP-301 had a significant radiosensitizing effect on OSCC cell lines, even CRR cells, compared with IR alone (Figures 2B and 2C). Calculation of the combination index demonstrated a synergistic antitumor effect of combination therapy in OSCC cell lines, including CRR cells (Figure 2D).

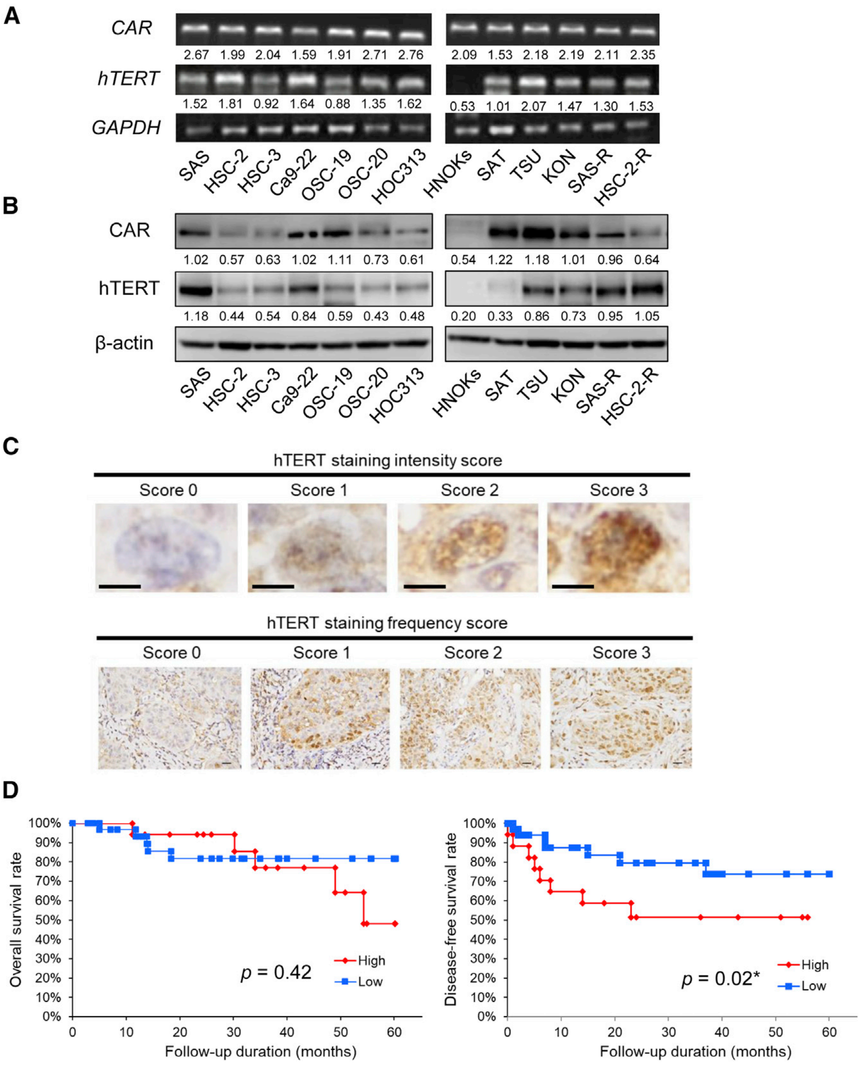

Figure 1. Expression analysis and clinicopathological significance of hTERT, which is important for the antitumor effect of OBP-301

(A and B) The mRNA and protein expression levels of CAR and hTERT in OSCC cell lines and HNOK. Cell lines cultured under identical conditions were harvested and utilized for real-time PCR and western blotting. Mean values obtained using an image analyzer (Figure S1) from at least three independent experiments are shown at the bottom of each band. (C) Determination of hTERT grade. Representative sections from the panels were scored for hTERT proportion and intensity as indicated. Scale bars, 15 μm (proportion) and 3 μm (intensity). The hTERT grade was the product of those scores (see materials and methods). (D) Overall survival (OS) and disease-free survival (DFS) of patients with OSCC based on the hTERT expression status. *$p < 0.05$. CAR, coxsackievirus and adenovirus receptor; HNOK, human normal oral keratinocytes; hTERT, human telomerase reverse transcriptase; OSCC, oral squamous cell carcinoma.

Regulation of apoptosis and autophagy may contribute to the radiosensitizing effect of OBP-301

We conducted a molecular biological study to elucidate the molecular mechanism underlying cell death caused by the combined use of OBP-301 and irradiation (IR). Apoptosis and autophagy are types of cell death induced after IR.[24] Therefore, the effects of OBP-301 on apoptosis and autophagy were investigated. The results of an annexin-V assay showed that radiation-induced apoptosis was significantly increased following combination treatment with OBP-301 and IR versus OBP-301 or IR alone (Figure 3A). In addition, the mitochondrial membrane potentials of the cell line irradiated after the administration of OBP-301 were significantly decreased versus those recorded after treatment with OBP-301 or IR alone (Figures 3B and 3C). Western blot analysis revealed a decrease in the phosphorylation of signal transducer and activator of transcription 3 (STAT3) and its downstream molecule, B cell lymphoma extra large (Bcl-xL) in the OBP-301 and IR groups compared with the IR alone group. A decrease in p62 and an increase in the light chain 3-II (LC3-II)/light chain 3-I (LC3-I) ratio are considered markers of autophagy.[31] In the present study, an increase in the LC3-II/LC3-I ratio and a decrease in p62 levels were observed in the OBP-301 and IR groups compared with the IR group. In addition, a slight increase in cleaved caspase 3 was observed in the OBP-301 and IR groups. Notably, significant changes in the expression of apoptosis/autophagy-related molecules that were not detected in the IR group were observed in the OBP-301 group (Figure 3D). In addition, changes in the expression of each molecule in the OBP-301 and IR groups were similar to those observed in the OBP-301 group. Furthermore, these phenomena were observed in two CRR cell lines, SAS-R and HSC-2-R.

Effect of OBP-301 combined with irradiation on the OSCC-CDX and CRR-CDX models

The therapeutic effects of OBP-301 combined with IR *in vivo* were determined using the OSCC-CDX and CRR-CDX models as shown in Figure 4A. In the OSCC-CDX model, PBS or BOP-301 alone failed to inhibit tumor growth; however, IR or OBP-301 combined with IR inhibited tumor growth. In addition, the combination of IR with OBP-301 significantly reduced tumor volume compared with IR or OBP-301 alone (Figure 4B). Furthermore, in the CRR-CDX model, an effect of OBP-301 and IR was observed compared with the OSCC-CDX model; however, the antitumor effect was decreased in the OBP-301 and IR alone groups at the end of the treatment schedule. Moreover, as observed in the OSCC-CDX model, IR plus OBP-301 exhibited the highest therapeutic effect (Figure 4C). In

Table 1. Correlation between hTERT expression and clinicopathological factors

Characteristic	Total	hTERT expression (n = 50 cases)		p value
		High, n (%)	Low, n (%)	
Age (years)				
Range	40–87	51–81	40–87	
≤65	15	5 (33.3)	10 (66.7)	0.948
>65	35	12 (34.3)	23 (65.7)	
Sex				
Male	28	10 (35.7)	18 (64.3)	0.773
Female	22	7 (31.8)	15 (68.2)	
cT category				
T2	20	5 (25)	15 (75)	0.548
T3, T4	30	12 (40)	18(60)	
cN category				
N0	14	7 (50)	7 (50)	0.136
≥N1	36	10 (27.8)	26 (72.2)	
cStage				
III	19	7 (36.8)	12 (63.2)	<0.001**
IV	31	10 (32.3)	21 (67.7)	
Differentiation				
Well-moderate	11	3 (27.3)	8 (72.7)	0.594
Poor	39	14 (35.9)	25 (64.1)	
Loco-regional recurrence				
Yes	17	9 (52.9)	8 (47.1)	0.023*
No	33	7 (21.2)	26 (78.8)	
Pathological response				
0, I, IIa, IIb	25	13 (52)	12 (48)	0.007**
III, IV	25	4 (16)	21 (84)	

Fisher's exact test was used to examine the relationships between hTERT expression and clinicopathologic factors. OSCC, oral squamous cell carcinoma; cT, clinical T stage; cN, clinical N stage; cStgae, clinical Stage.
*p < 0.05 and **p < 0.01.

immunohistochemical analyses, the TdT-mediated dUTP-biotin nick-end labeling (TUNEL) assay showed a significant increase in apoptotic cells in the IR plus OBP-301 group (Figures 5A and 5C), whereas autophagy was also significantly enhanced by immunohistochemical staining analysis as measured by a decrease in p62 expression (Figures 5B and 5D) in both models.

Effect of combination therapy with OBP-301 and IR on the PDX model

To evaluate the effects of combination therapy with OBP-301 and IR, we conducted experiments using a PDX model based on the schedule used in human clinical studies (Figure 6A).[16] As shown in Figure 6B, IR combined with OBP-301 significantly reduced tumor volume versus IR monotherapy in the PDX model. Moreover, immunohistochemical staining of samples collected from PDX tumors at the end of treatment showed that the combination of IR plus OBP-301 was associated with an increase in the number of apoptotic and autophagy cells compared with IR monotherapy (Figure 6C).

DISCUSSION

In this study, we examined *in vitro* and *in vivo* whether combination therapy with OBP-301 and radiation may be a new treatment option for patients with OSCC in whom the disease is refractory to standard treatment. Initially, we analyzed the expression of hTERT, an important molecule for OBP-301 to exert its effects *in vivo*. The results showed that hTERT was expressed in almost all OSCC cell lines, including CRR cell lines (Figures 1A and 1B). Also, a certain level of hTERT expression was observed in a majority of human clinical specimens (Figure 1C). In addition, our clinicopathological analyses revealed that OSCC with high hTERT expression is refractory to existing therapies and potentially represents a patient population in whom disease control may be difficult (Tables 1 and 2 and Figure 1D). Aberrant expression of hTERT has been reported in numerous malignancies.[29] Moreover, hTERT expression is closely associated with tumorigenesis, maintenance of cancer cell stemness, cell proliferation, inhibition of apoptosis, evasion of senescence, and metastasis.[30,32,33] Taken together, in line with previous findings, our initial findings suggest that OSCC with high hTERT expression may be resistant to existing therapies (e.g., chemotherapy and radiotherapy); thus, it may also be a good therapeutic target for OBP-301. However, there are various cell death pathways initiated by OBP-301, and its antitumor effect may occur through more than one of them. Therefore, CAR and hTERT may not be the only molecules that determine the antitumor effect of OBP-301. In the future, we will identify the specific cell death pathway(s) depending on the tumor type, and consider combination therapy based on CAR and hTERT expression levels.

In previous studies, the antitumor effect of OBP-301 as a single agent has been confirmed in various carcinomas, including HNSCC.[14,18,25,34–39] Consistent with previous studies, in the present study, we were able to confirm the concentration-dependent antitumor effect of OBP-301 as a single agent in several OSCC cell lines, including CRR cells (Figure 2A). We also confirmed that the synergistic effect of OBP-301 and IR in OSCC cell lines is stable under the condition of multiplicity of infection (MOI) of 10^4 viral particles (vp)/cell and an IR dose of >2 Gy. Interestingly, this effect was also observed in CRR cells (Figures 2B–2D). Previously, the phenomenon of enhanced antitumor efficacy in combination with OBP-301 was reported in chemotherapy,[13,40] radiotherapy,[25] and immunotherapy[26] in various malignancies. In HNSCC, including OSCC, it has been reported that OBP-301 exerts favorable antitumor effects in combination with chemotherapy.[20] After comparing the radiosensitivity of multiple HNSCC cell lines, Takahashi et al.[22] reported that radiation therapy with OBP-301 may be effective in cell lines that are considered to have relatively low radiosensitivity. In this study, we also obtained similar results. CRR cells are useful in radioresistance studies.[5] In this study, these cells allowed us to accurately assess the potential of OBP-301 to contribute to overcoming radioresistance in radioresistant OSCC.

Table 2. Multivariate regression analysis results for predicting disease-free survival in 50 patients with OSCC

Variable	Assigned score	Hazard ratio (95% CI)	p value
Clinical T category			
T2	0	0.659 (0.195–2.278)	0.502
T3, T4	1		
Clinical N category			
N0	0	0.880 (0.206–3.946)	0.862
≥N1	1		
Clinical stage			
III	0	2.378 (0.727–9.258)	0.157
IV	1		
Differentiation			
Well-moderate	0	1.222 (0.368–5.531)	0.760
Poor	1		
Worst pattern of invasion			
1[a], 2[b], 3[c]	0	2.908 (0.867–10.112)	0.083
4[d], 5[e]	1		
Pathological response			
Grade 0, I, II	0	1.114 (0.375–3.047)	0.838
Grade ≥III	1		
hTERT expression status			
High expression	0	3.241 (1.112–9.992)	0.031*
Low expression	1		

CI, confidence interval; OSCC, oral squamous cell carcinoma.
*$p < 0.05$.
[a]Broad pushing margin.
[b]Broad finger-like projections or separate large islands.
[c]Invasive islands (>15 cells).
[d]Islands of <5 cells, strands of tumor cells or single-cell infiltration.
[e]Tumor satellites separated from the main tumor interface by >1 mm.

We focused our analyses on the various types of cell death associated with IR exposure (data not shown). As a result, enhanced apoptosis and autophagy following treatment with the combination of OBP-301 and IR were observed to varying degrees in *in vitro* and *in vivo* settings (Figures 3A–3D, 5A–5D, and 6C). In esophageal, gastric, and lung cancers, OBP-301 inhibited the MRE11-RAD50-NBS1 (MRN) complex, resulting in radiosensitization and enhanced induction of apoptosis.[21] In soft tissue sarcomas, OBP-301 combined with radiotherapy enhanced apoptosis through the suppression of MCL1.[25] In neuroblastoma, it was reported that monotherapy with OBP-301 induced autophagy-related cell death.[14] Kuwahara et al. suggested that the suppression of autophagy may be associated with the reduced radiosensitivity of radioresistant cells.[24] Based on the results of previous reports, our data suggest that OBP-301 may contribute to overcoming radioresistance in CRR cells by inducing varying degrees of apoptosis, primarily through autophagy. Moreover, OBP-301 replicated more efficiently in combination therapy with radiation compared with OBP-301 alone (Figures S5A–S5E). Ishikawa et al. reported that the replication efficiency of OBP-301 was enhanced in combination with paclitaxel.[41] In OSCC, radiotherapy may also be closely associated with the therapeutic effect because OBP-301 is efficiently replicated, which suggests that it is an effective combination therapy.

In addition to the above results that support previous findings, we also found that downregulation of Bcl-xL following decreased STAT3 phosphorylation may be involved in the regulation of apoptosis and autophagy by OBP-301 (Figure 3D). According to a growing body of evidence, STAT3 is an important molecule involved in tumorigenesis and tumor development, as well as the occurrence of chemoresistance and radioresistance via the transcriptional regulation of several apoptosis- and autophagy-related genes, such as members of the Bcl2 family (including Bcl-xL).[42,43] Bcl-xL localizes to the mitochondrial outer membrane, preventing loss of mitochondrial membrane potential and inhibiting apoptosis.[44,45] Bcl-xL forms a complex with beclin-1 (BECN1), an autophagy-promoting factor, and suppresses autophagy; however, disassociation of BECN1 by Bcl-xL suppression promotes autophagy.[46] Our present data suggest that regulation of the STAT3-Bcl-xL pathway may contribute to the radioresistance of radioresistant OSCC through apoptosis and autophagy. However, further investigation is warranted to validate this hypothesis.

Subcutaneous and orthotopic xenograft tumor models using human cancer cell lines are frequently employed for the *in vivo* evaluation of therapeutic potential.[47] Therefore, subcutaneous inoculation models have been used in previous studies on OBP-301.[9,13,14,34,48] We confirmed the *in vivo* antitumor effect of radiation therapy with OBP-301 using a subcutaneous parental and radioresistant OSCC-CDX tumor model (Figure 4B). Moreover, we also confirmed markedly better antitumor effects than those observed with radiation therapy alone in the PDX model (Figure 6B). This is a more useful model than the CDX model for evaluating the effects of therapies in translational research.[49] In the PDX model, both IR and OBP-301 with IR showed better than expected antitumor effects in the PDX model compared with the CDX model. This may have occurred because the tumors used in the PDX model were radiosensitive compared with the CDX model. Notably, TUNEL assays and immunohistochemical analyses of the residual tumors from the OSCC-CDX, CRR-CDX, and PDX models revealed significant changes in apoptosis and autophagy indicators that supported the changes observed *in vitro* (Figures 5A–5D and 6C). Collectively, these data indicate that the therapeutic effect of OBP-301 combined with radiotherapy and the underlying mechanism involved in overcoming radioresistance are consistent *in vitro* and *in vivo*. Nonetheless, Kanaya et al. reported that OBP-301 effectively potentiates the antitumor effect of PD-1 antibodies and the abscopal effect by inducing immunogenic cell death (ICD)-related molecules in an allogeneic transplantation model.[26] In our preliminary experiments, the expression of representative ICD-related molecules, such as HMGB1 and calreticulin, was decreased in CDX models treated with OBP-301 alone or IR combined with OBP-301 compared with IR alone (Figure S6). This may be related to the effective induction of autophagy by OBP-301. Although it is difficult to evaluate the abscopal effect, which is closely

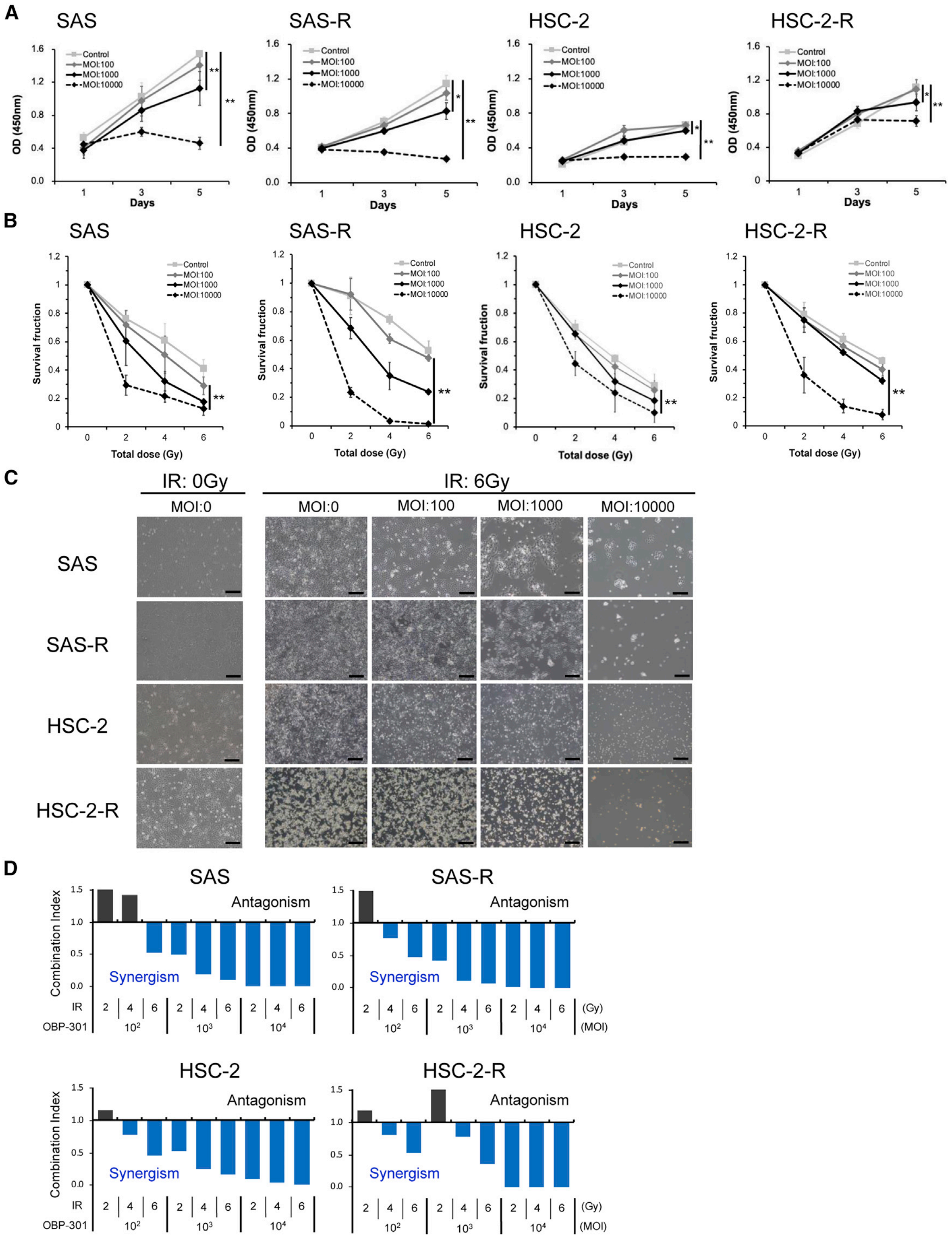

Figure 2. Radiosensitizing effect of OBP-301 in clinically relevant radioresistant OSCC cell lines

(A) The proliferation of OSCC cell lines (SAS, SAS-R, HSC-2, HSC-2-R) untreated (control) or treated with stepwise concentrations of OBP-301 (MOI: 10^2, 10^3, and 10^4 vp/cell) was examined using the WST assay after 1, 3, and 5 days. (B) Graphs showing the results of the MHDS assay. OSCC cell lines were treated with stepwise concentrations of OBP-301 (MOI: 0 [control], 10^2, 10^3, and 10^4 vp/cell) and irradiated with 0, 2, 4, and 6 Gy. These results are shown as the means of at least three independent experiments performed in triplicate; *$p < 0.05$ and **$p < 0.01$. (C) Representative images captured prior to performing the MHDS assay. OSCC cells were treated with OBP-301 (MOI: 0, 10^2, 10^3, and 10^4 VP/cell) and irradiated with 6 Gy. (D) The combination index was calculated using the CalcuSyn software (BioSoft, Inc., Cambridge, UK). Interaction indices <1 and >1 denoted synergy and antagonism, respectively. MHDS, modified high-density survival assay; MOI, multiplicity of infection; OSCC, oral squamous cell carcinoma; vp, viral particles.

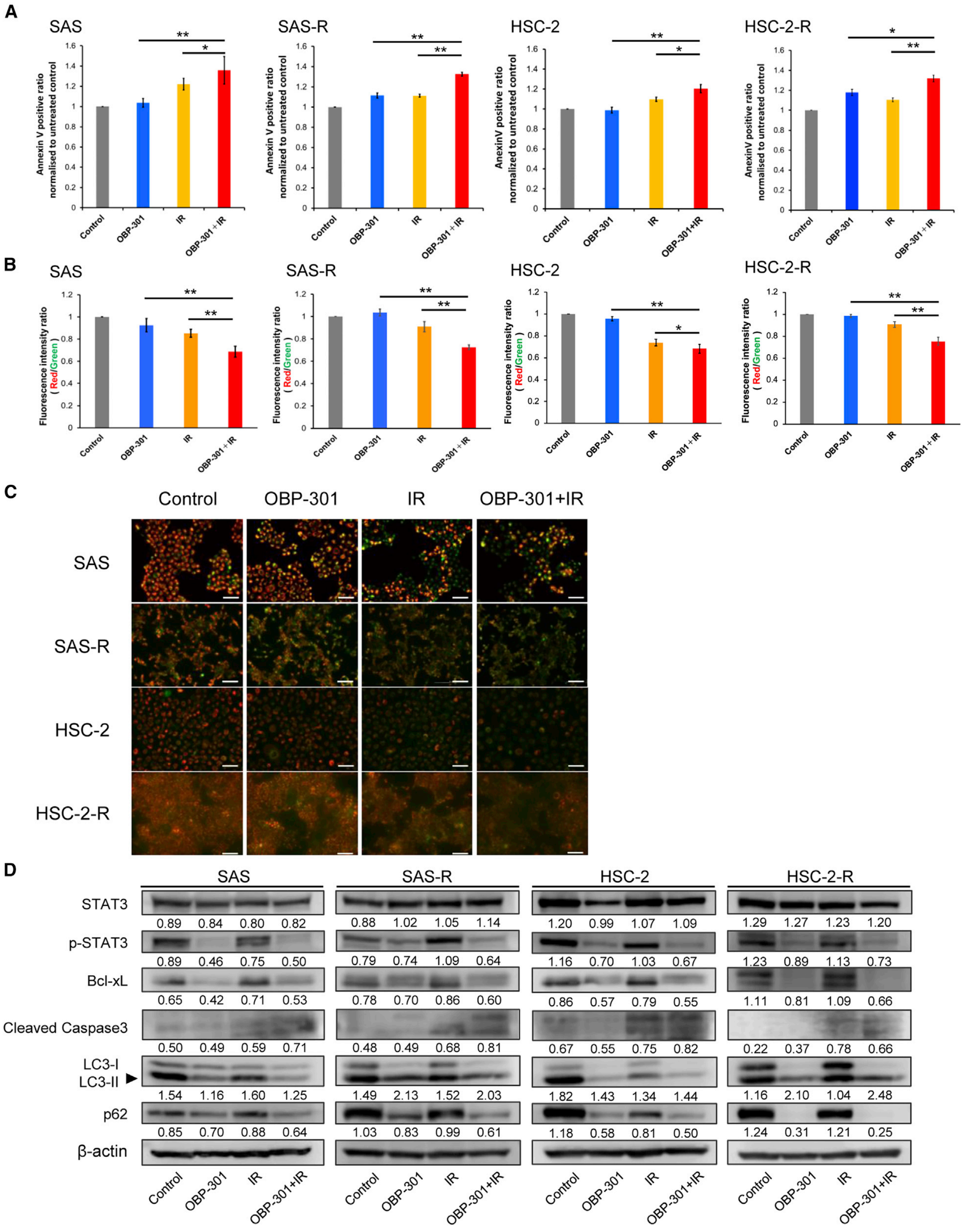

(legend on next page)

related to acquired immunity, in the xenograft model used in the present study, it is necessary to verify whether OBP-301 combined radiotherapy exhibits a local antitumor effect as well as a secondary therapeutic effect through antitumor immunity in an allogeneic OSCC model.

Currently, cisplatin and cetuximab are the drugs used in the combination of radiotherapy and chemotherapy for the treatment of HNSCC; the usefulness of these agents has been examined by reports on their efficacy and comparative studies.[50,51] However, cisplatin is associated with various side effects, such as drug resistance and renal impairment,[52] whereas cetuximab is linked to a risk of serious allergic reactions after the first dose.[53] Therefore the patient's general condition often forces the use of radiotherapy alone. Thus far, there are no reports of serious side effects of OBP-301 in clinical trials.[15,16] Consistently, in the present *in vivo* study, we did not observe any serious adverse effects of OBP-301 in vital organs, thereby confirming the safety of this treatment (Figure S4). Ultimately, the combination of OBP-301 and radiotherapy may be a relatively safe and easy to use therapeutic modality for patients with advanced OSCC and limited treatment options in various backgrounds.

Takahashi et al. identified the radiosensitivity of several HNSCC cell lines, which they defined as relative radioresistance. Their study reported that OBP-301 exerts its sensitizing effect through the DNA repair pathway.[22] Similarly, we had reported that OBP-301 exerts its radiosensitizing effect in soft-tissue sarcomas by inhibiting the antiapoptotic protein MCL1.[25] However, the following points are considered novel. First, we used CRR cells established from the same cell type rather than comparing different cell lines. Second, the enhancement of autophagic cell death by OBP-301 has also been implicated, and we observed that STAT3 phosphorylation may be involved in the upstream enhancement. Third, to confirm the practicality of OBP-301 in clinical practice, we conducted experiments with several cells, including CRR cells and PDX, consistent with routine clinical practice. Although further studies are needed for some of these issues, we believe that the results provide a rationale for the development of new treatments for radioresistant OSCC.

In summary, the present study demonstrated that OBP-301 combined with radiotherapy may be a novel treatment option for radioresistant OSCC. In addition, the STAT3-Bcl-xL axis may regulate apoptosis and autophagy as a new molecular mechanism involved in radiosensitization induced by OBP-301 in OSCC (Figure 7). These findings provide evidence to promote the development of novel treatment strategies using OBP-301 for patients with refractory OSCC or those who are intolerant to existing standard treatment.

MATERIALS AND METHODS

Cell lines

Human OSCC cell lines derived from oral cancer (SAS, HSC-2, HSC-3, Ca9-22, OSC-19, OSC-20, SAT, and KON) were purchased from the National Institute of Biomedical Innovation (Osaka, Japan). The HOC-313[54] and TSU[55] cell lines were kindly provided by Professor Kawashiri (Kanazawa University). HNOK cells were purchased from the American Type Culture Collection (ATCC; Manassas, VA, USA; PCS-200-014). SAS-R and HSC-2-R, which were established from SAS and HSC-2 cells, were used as the CRR cell lines. The CRR cell lines were produced by exposing cells to gradually increasing X-ray doses.[5] The OSCC cell lines were cultured in Dulbecco's modified Eagle medium (DMEM; D6429; Sigma-Aldrich, Saint Louis, MO, USA) supplemented with 10% fetal bovine serum (Sigma-Aldrich) at 37°C and 5% CO_2. HNOK cells were cultured in dermal cell basal medium (PCS-200-030; ATCC) supplemented with the Keratinocyte Growth Kit (PCS-200-040; ATCC). CRR cells continued to proliferate under a daily IR dose of 2 Gy for >30 days *in vitro*.

IR

IR doses of 2, 4, 6, and 10 Gy were delivered using a 150 KVp X-ray generator with total filtration through a 0.5-mm aluminum plUS 0.1-mm copper filter (MBR-1520R; Hitachi, Tokyo, Japan). The dose rate (1.01 Gy/min) was measured using a thimble ionization chamber (IC 17A; Far West Technology, Goleta, CA, USA).

Measurement of cell proliferation activity

OSCC cell lines (SAS, HSC-2) and CRR cell lines (SAS-R, HSC-2-R) in the logarithmic growth phase were seeded in 96-well microplates (2.5×10^3 cells per well). At 24 h after seeding, cells were incubated with OBP-301 (MOI 0, 10^2, 10^3, and 10^4 vp/cell). Every 24 h after incubation, Cell Counting Kit-8 (Dojindo, Kumamoto, Japan) was added to each well, and the color reaction was carried out for 1 h. The absorbance at 450 nm was measured using a microplate reader (iMark microplate reader; Bio-Rad Laboratories, CA, USA).

Figure 3. Enhancement of apoptosis and autophagy contributes to the radiosensitizing effect of OBP-301

(A) Graphs showing a comparison of the annexin-V-positive ratio of the combination therapy with OBP-301 and IR versus OBP-301 or IR monotherapy in OSCC cell lines (SAS, SAS-R, HSC-2, HSC-2R). The measurement was performed 48 h after IR. OBP-301 MOI 10^3 vp/cell; IR 10 Gy. (B and C) Graphs comparing the change in mitochondrial membrane potential (MitoMP) for the combination therapy with OBP-301 and IR versus OBP-301 or IR monotherapy in OSCC cell lines (SAS, SAS-R, HSC-2, HSC-2R). Representative immunofluorescence images are shown in (C). Red and green fluorescence denotes high and low MitoMP, respectively. These graphs and images were obtained at 12 h after IR. OBP-301 MOI 10^3 vp/cell; IR 10 Gy. These results are shown as the means of at least three independent experiments performed in triplicate; *$p < 0.05$ and **$p < 0.01$. (D) Western blots of apoptosis and autophagy molecular components (p-STAT3, Bcl-xL, cleaved caspase 3, LC3-I/II, and p62) in SAS, SAS-R, HSC-2, and HSC-2-R cells at 48 h after 6 Gy IR. The expression of β-actin was used as an internal control. Bcl-xL, B cell lymphoma extra large; OSCC, oral squamous cell carcinoma; p-STAT3, phosphorylated signal transducer and activator of transcription 3; PARP, poly(ADP-ribose) polymerase; IR, irradiation; MOI, multiplicity of infection. Mean values obtained using an image analyzer (Figure S2) from at least three independent experiments are shown at the bottom of each band.

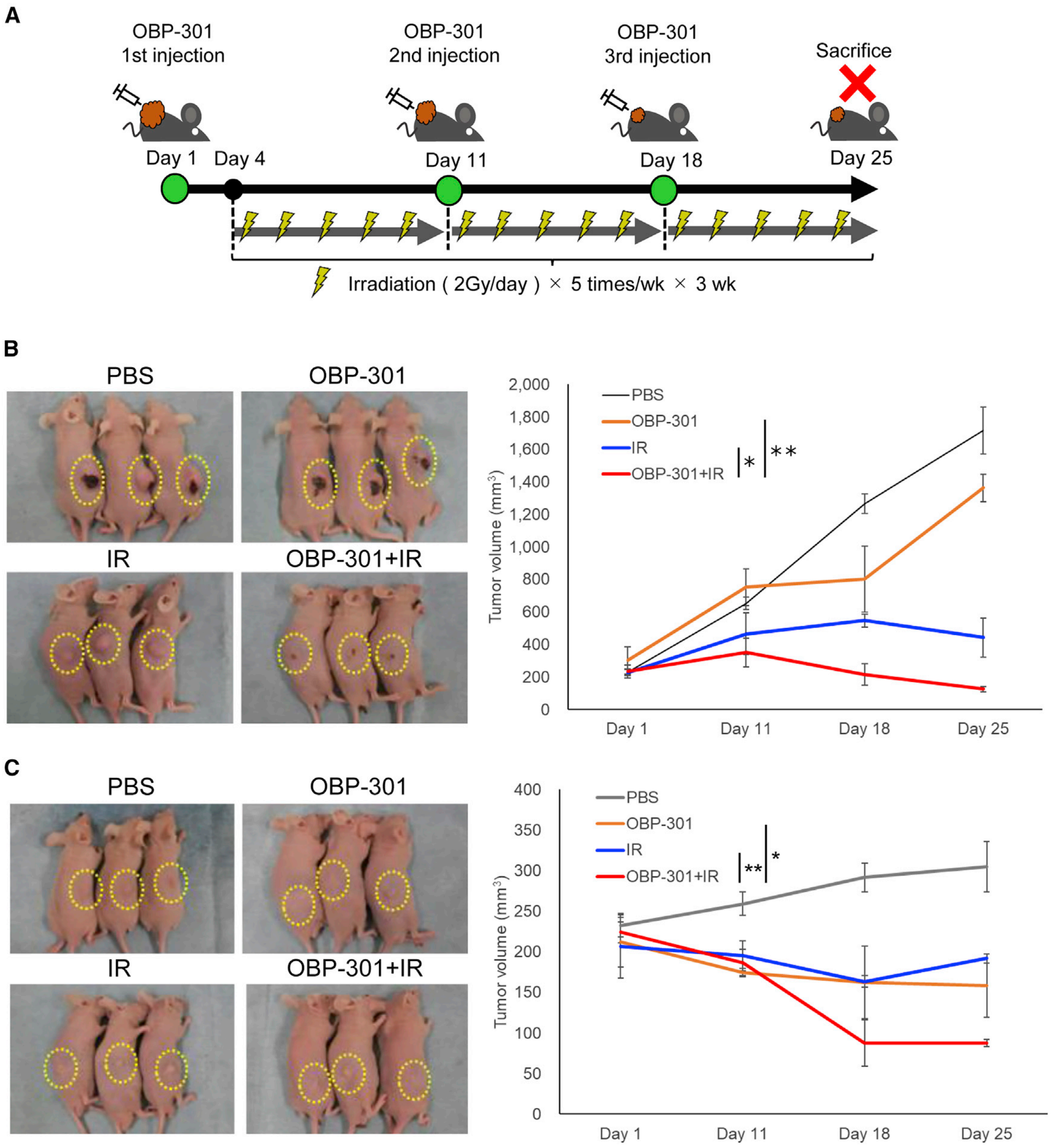

Figure 4. Effects of combination therapy with OBP-301 and irradiation (IR) in the xenograft mouse model

(A) The experimental protocol of the xenograft mouse model described in the materials and methods. The experiment was initiated when the diameter of the transplanted tumor reached 7–10 mm. OBP-301 (1×10^{11} vp/100 μL) was locally injected on days 1, 11, and 18 of treatment. IR was initiated on day 4 of treatment and 2 Gy was administered five times per week for 3 weeks. (B and C) Images of the (B) SAS and (C) SAS-R xenograft mouse models after treatment PBS, OBP-301 monotherapy, IR monotherapy (30 Gy) and the combination of IR (30Gy) plus OBP-301. The graphs of the tumor volume transition are shown. The mean ± SD of three independent experiments was calculated; n = 3 per group; $^{*}p < 0.05$ and $^{**}p < 0.01$. vp, viral particles.

MHDS assay

MHDS assay was performed as previously reported.[5] Exponentially proliferating cells (1×10^6) were seeded into 60-mm dishes (AGG, Tokyo, Japan) and cultured in DMEM supplemented with 10% fetal bovine serum for 24 h. Cells were treated with OBP-301 (MOI 0, 10^2, 10^3, 10^4 vp/cell) and irradiated (0, 2, 4, 6 Gy) after 24 h. At 72 h after IR, 10% of the cells in each dish were seeded into a new 60-mm dish and incubated for another 72 h. The total number of cells in each dish was determined using a cell counter (Bio-Rad Laboratories), and the cell viability was calculated.

Combination index analysis

The combinatory effect of OBP-301 and IR was analyzed by calculating the Combination Index using the CalcuSyn software

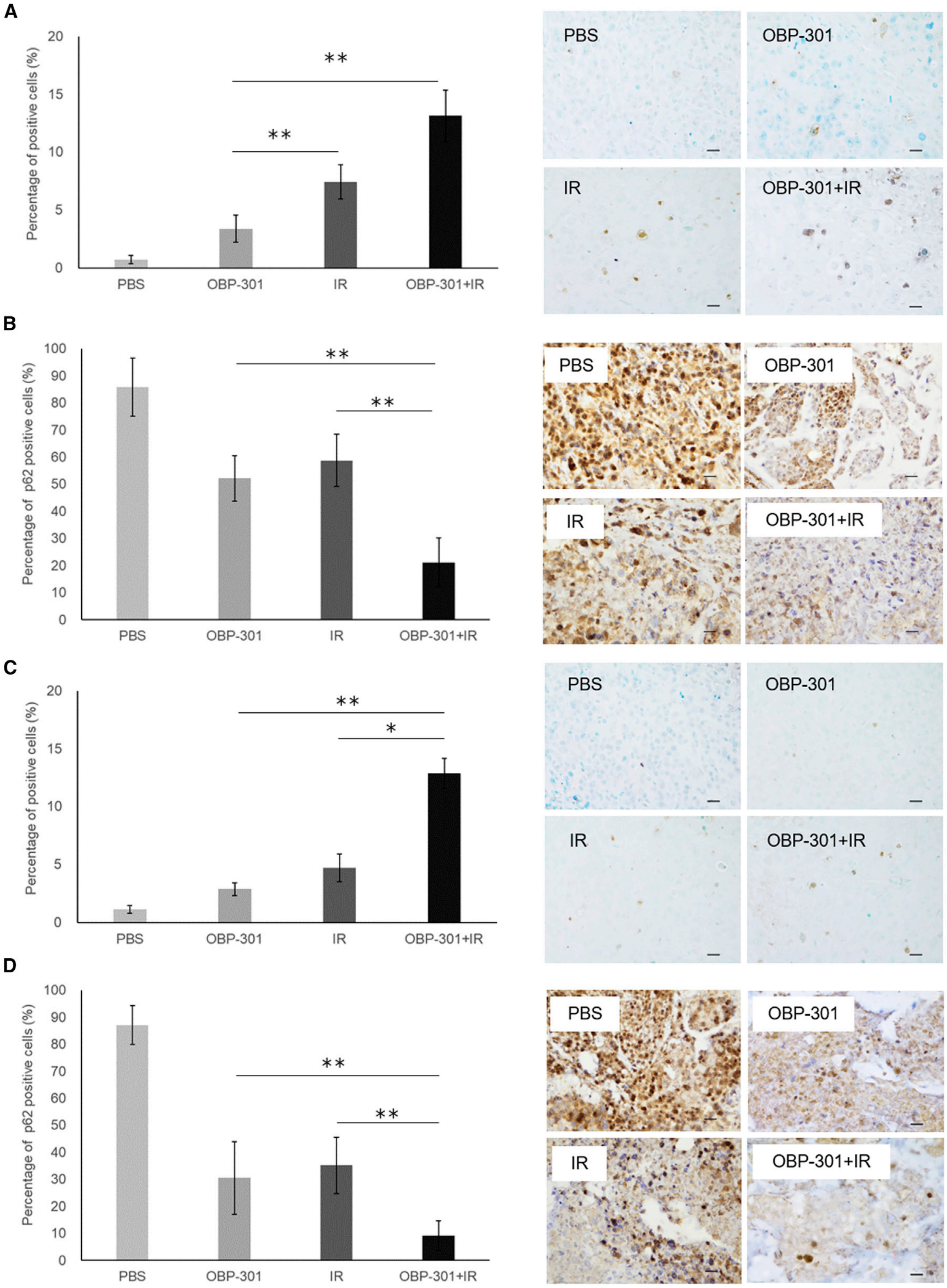

(legend on next page)

(BioSoft, Cambridge, UK). The calculation of the Combination Index was based on the method previously described by Chou.[56]

Western blotting

Whole-cell proteins were extracted using Minute Cytoplasmic and Nuclear Extraction Kits (Invent Biotechnologies, Plymouth, MN, USA). Total cell protein (5 μg) was separated by 10%–20% sodium dodecyl sulfate-polyacrylamide gel electrophoresis and transferred to nitrocellulose membranes. The membrane was subjected to blocking treatment for 60 min and incubated with a primary antibody cocktail (diluted in Tris-buffered saline with Tween 20 containing 5% bovine serum albumin) overnight at 4°C. A list of the antibodies used in this study is shown in Table S1. Subsequently, the membrane was washed with Tris-buffered saline with Tween 20 and incubated with a secondary antibody cocktail for 60 min at room temperature. The membrane was washed and visualized using the ECL Prime Detection Kit (GE Healthcare, Chicago, IL, USA). The chromogenic light was measured using a C-Digit blot scanner and the images were analyzed using C-Digit's Image studio (LI-COR Biosciences, Lincoln, NE, USA). The relative expression of each protein was determined using ImageJ 1.52q software (National Institutes of Health, Bethesda, MD, USA).

RT-PCR

Total RNA was isolated using the FastGene RNA Basic Kit (NIPPON Genetics, Tokyo, Japan) and reverse transcribed into cDNA using the ReverTra Ace qPCR RT Kit (Toyobo, Osaka, Japan). PCR was performed using the Thunderbird SYBR qPCR Mix (Toyobo). A list of the primers used in this study is shown in Table S2. The PCR products were analyzed using agarose gel electrophoresis and visualized using staining with ethidium bromide. The relative expression of each gene was determined using ImageJ 1.52q software (National Institutes of Health, Bethesda, MD, USA).

Clinical specimens

For the clinicopathological analyses, pretreatment tissue samples were obtained from 50 patients with locally advanced OSCC who underwent preoperative CRT at the Kumamoto University Hospital (Kumamoto, Japan) between October 2003 and January 2009. We excluded human papillomavirus-positive tumors from the analysis based on the immunostaining results for p16 (a surrogate marker for human papillomavirus infection). All 50 patients were enrolled in our phase II study[57] and underwent curative surgery following preoperative CRT. The preoperative CRT was conducted as previously described.[57] The staging and determination of tumor differentiation were performed according to the 7th edition of the *Cancer Staging Manual* of the American Joint Committee on Cancer.[58] The pathological response to CRT was graded using specimens obtained during surgery based on the criteria proposed by Shimosato et al.,[59] as follows: grade I, no destruction of tumor structures; grade IIa, mild destruction of the tumor structure (i.e., "viable tumor cells" are frequently observed); grade IIb, severe destruction of the tumor structure (i.e., "viable tumor cells" are few); grade III, presence of nonviable tumor cells; and grade IV, absence of tumor cells. This study was approved by the Ethics Committee of Kumamoto University (approval no. 174) and conducted in accordance with the guidelines of the Declaration of Helsinki.

Immunohistochemical staining analysis

Formalin-fixed, paraffin-embedded specimens prepared from patients with OSCC and samples obtained from mouse experiments were thinly sliced into 4-μm sections and adhered and fixed on MAS-GP-coated slides (Matsunami Glass, Osaka, Japan). Following deparaffinization and rehydration with ethanol, the sections were treated with methanol containing 3% hydrogen peroxide for 30 min to remove endogenous peroxidase activity. The sections were subsequently reacted with Protein Block Serum-Free reagent (Dako, Glostrup, Denmark) for 15 min. A list of antibodies used in this study is shown in Table S3. All specimens were contrast stained with hematoxylin for 1 min prior to dehydration and inclusion. The level of hTERT expression was determined based on the system introduced by Allred et al.[60] We semi-quantified the proportion of hTERT-positive cells among the total number of cancer cells and the staining intensity for hTERT. The proportion score of hTERT-positive cells was classified as follows: 0, <1%; 1, 1%–10%; 2, 11%–50%; and 3, >50%. The intensity score was classified as follows: 0, lack of intensity; 1, weak, detectable only in high-power fields; 2, moderate, detectable in low-power fields; and 3, strong. The proportion and intensity scores were summed to produce an hTERT score ranging from 0 to 6. Values of 0–4 and 5–6 denoted low and high hTERT scores, respectively. The scoring was conducted by two examiners, who were blinded to the clinicopathological data. p62 expression was determined by counting the number of positive cells of 100 in five random fields (400× objective). A percentage of positive cells was calculated by dividing the number of positive cells by the total number of cells per sample and multiplying by 100. The antibodies used in above analyses are shown in Table S3.

Figure 5. Analysis of apoptosis and autophagy *in vivo*

(A and B) The results in the OSCC-CDX model using SAS. (A, left) The number of apoptotic cells obtained from three independent experiments was calculated and statistically analyzed. The results are shown as means ± SD of three independent experiments; $^{**}p < 0.01$. (A, right) Representative images of the TdT-mediated dUTP-biotin nick-end labeling (TUNEL) assay. Scale bars: 20 μm. (B, left) The number of autophagic cells obtained from three independent experiments was calculated and statistically analyzed. The results are shown as means ± SD of three independent experiments; $^{**}p < 0.01$. (B, right) Representative images of p62 immunostaining. Scale bars: 20 μm. (C and D) The results in the CRR-CDX model using SAS-R. (C, left) The number of apoptotic cells obtained from three independent experiments was calculated and statistically analyzed. The results are shown as means ± SD of three independent experiments; $^{*}p < 0.05$, $^{**}p < 0.01$. (C, right) Representative images of the TUNEL assay. Scale bars: 20 μm. (D, left) The number of autophagic cells obtained from three independent experiments was calculated and statistically analyzed. The results are shown as means ± SD of three independent experiments; $^{**}p < 0.01$. (D, right) Representative images of p62 immunostaining. Scale bars: 20 μm.

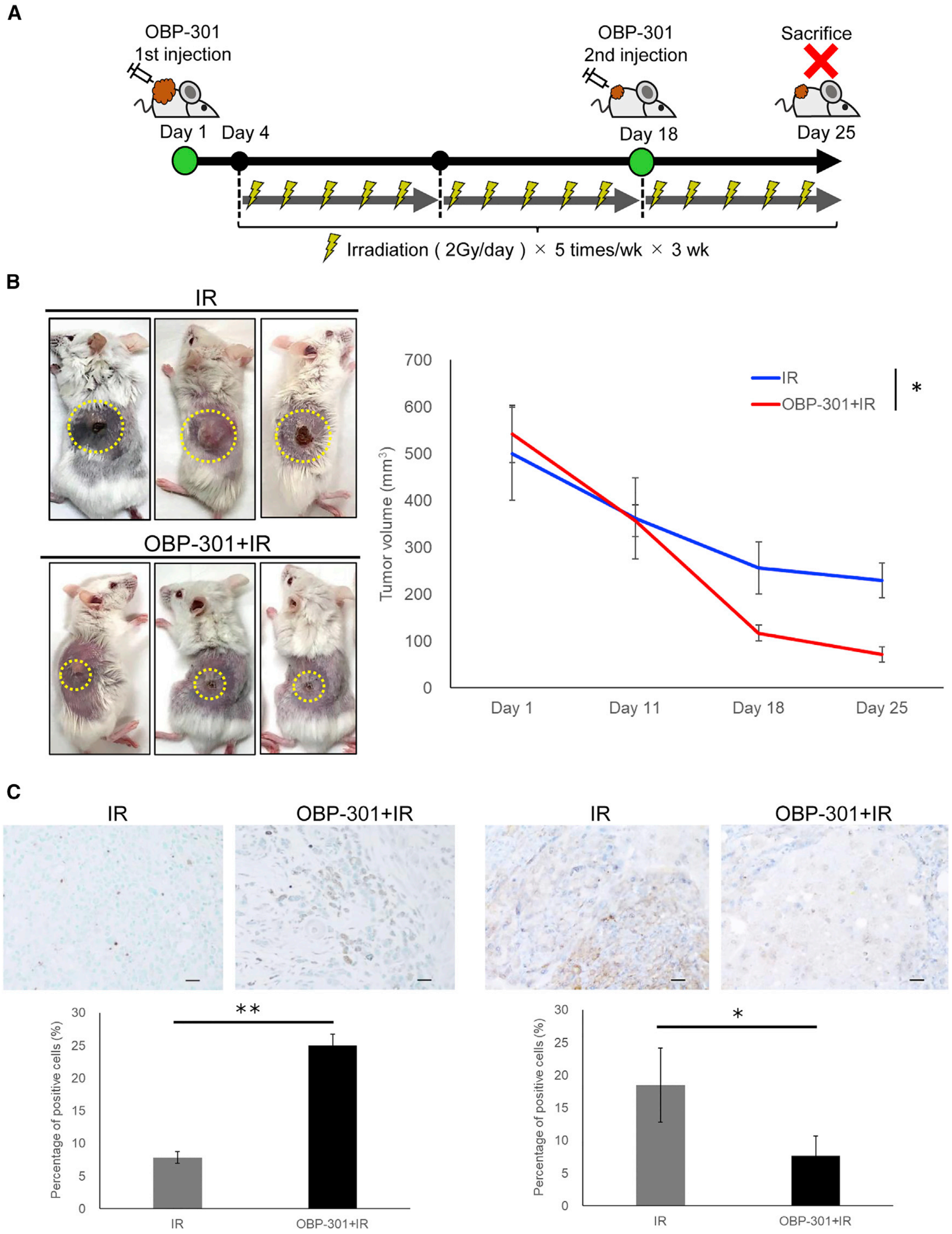

Figure 6. Effect of combination therapy with irradiation (IR) and OBP-301 in the PDX model

(A) The experimental protocol of the PDX model described in the materials and methods. The experiment was initiated when the diameter of the transplanted tumor reached 10–15 mm. OBP-301 (1×10^{11} vp/100 μL) was locally injected on days 1, 11, and 18 of treatment. IR was initiated on day 4 of treatment and 2 Gy was administered five times per week for 3 weeks. (B) Images of the PDX model after treatment with IR monotherapy (0–30 Gy) and the combination of OBP-301 plus IR (left). The graphs of the tumor

(legend continued on next page)

Mitochondrial membrane potential assay

The mitochondrial membrane potential (MitoMP) was measured using the JC-1 MitoMP detection kit (Dojindo). JC-1 aggregates generated red fluorescence, indicating a normal function for MitoMP. In contrast, the JC-1 monomer form generated green fluorescence, indicating dysfunction for MitoMP. After each experimental manipulation, cells were treated with JC-1 (4 μM, 37°C, 30 min). Finally, the MitoMP level was analyzed by calculating the fluorescence intensity ratio (red, ex/em 535/595 nm; green, ex/em 485/535 nm) using a fluorescence plate reader (SpectraMax i3x; Molecular Devices, San Jose, CA, USA).

Annexin-V apoptosis assay

Cell lines (SAS, HSC-2, SAS-R, HSC-2-R) were treated with the Annexin V Apoptosis Assay (Promega, Madison, WI, USA) 24 h after seeding in 96-well plates (100 μL per well), and the cells were divided into four groups (control, OBP-301, IR, OBP-301 + IR). After treatment of each group, the fluorescence intensity (ex/em 485/525 nm) was measured using a plate reader (SpectraMax i3x; Molecular Devices) 72 h after IR.

OSCC-CDX and CRR-CDX models

BALB/c nu/nu mice (BALB mice; Charles River Laboratories Japan, Kanagawa, Japan) were used in this study. The animals were bred at the Kumamoto University Animal Resource Development and Research Facility, and the experiments were conducted in accordance with the ethical standards for animal experiments at Kumamoto University. SAS and SAS-R cells were detached by treatment with trypsin, washed in DMEM without serum, and resuspended in phosphate-buffered saline (PBS) at a concentration of 1×10^7 cells/100 μL. The cell suspension was transplanted into the dorsal subcutaneous region of the BALB mice (100 μL per animal). The treatment experiment was initiated when the longest diameter of the tumor reached 9–10 mm. After completion of the treatment, the mice were euthanized by administration of ether, and the tumors were removed. Tumor tissues were fixed with 10% buffered formalin for immunohistochemical staining and paraffin-embedded to prepare blocks for paraffin sections.

PDX model

The PDX model was established by transplanting excised tissue from a patient with tongue cancer (detailed clinical data are shown in Figure S3) treated in our department directly into the back of BALB/c-Rag2/Jak3 knockout mice. The treatment experiment was initiated when the tumor reached 10–15 mm in diameter.

Treatment schedule in the mouse model

The treatment experiments in the mouse model were planned based on a phase I study by Shirakawa et al. for a clinical application.[16] In the OSCC- and CRR-CDX models, OBP-301 (1×10^{11} vp/100 μL) was injected locally into the tumor on days 1, 11, and 18 of treatment. IR was initiated on day 4 of treatment, in which 2 Gy was administered five times per week for a total of 3 weeks. The PBS-treated group was used as the experimental control, and the OBP-301 alone group was also used for comparison. In the PDX model, the treatment was conducted exactly the same way as in the clinical study being conducted, and OBP-301was injected locally into the tumor on days 1 and 18 of treatment. Following the ethical rules for animal experimentation at our institution, the total IR dose was set at 30 Gy. Based on the experimental results in OSCC-CDX and the preliminary results of OBP-301 monotherapy in the PDX model (Figure S7), the PDX-based treatment studies compared only IR and IR combined with OBP-301. Tumor volume was determined by measuring the length and width of the tumor using calipers (n = 3 per group). Tumor volume was calculated using the following formula: V (volume) = $L \times W \times W \times 0.5$ (L represents the length of each tumor; W represents the width of each tumor).

TdT-mediated dUTP-biotin nick-end labeling assay

Apoptosis in tissue samples obtained from the OSCC-CDX, CRR-CDX, and PDX model was measured using the *In Situ* Apoptosis Detection Kit (TaKaRa) according to the manufacturer's protocol.

Ethical approval

This study was approved by the Ethics Committee of Kumamoto University (approval no. 2389, 1427) and performed in accordance with Good Clinical Practice and the Declaration of Helsinki guidelines. This study was approved by the Institutional Animal Care and Use Committee (permission nos. A30-086 and A2022-086) and carried out according to the Kumamoto University Animal Experimentation Regulations.

Statistical analysis

Differences in the means between two groups were analyzed using the Wilcoxon rank-sum (Mann-Whitney) test. Differences in the means between multiple groups were analyzed by one-way analysis of variance using the Bonferroni/Dunn test. The OS and DFS were defined as the time from the initiation of CRT treatment to the date of death by any cause and the date of recurrence of cancer or death by any cause, respectively. The Kaplan-Meier method was used to estimate the probability of OS and DFS as a function of time, and statistical differences in the survival of patients in subgroups were compared using the log-rank test. All p values were calculated based on two-tailed statistical analysis, and $p < 0.05$ denoted statistically significant difference. Statistical analysis was performed using the JMP 9 software program (SAS Institute, Cary, NC, USA).

volume transition are shown (right). The mean ± SD of three independent experiments was calculated; n = 3 per group; *p < 0.01. (C) (Top) Representative images of the TUNEL assay and p62 immunostaining using samples obtained from the PDX model after the experiment (IR monotherapy and the combination of OBP-301 plus IR therapy). Scale bars: 20 μm. (Bottom) The number of positive cells obtained from three independent experiments was calculated and statistically analyzed. The results are shown as means ± SD of three independent experiments; *p < 0.05, **p < 0.01.

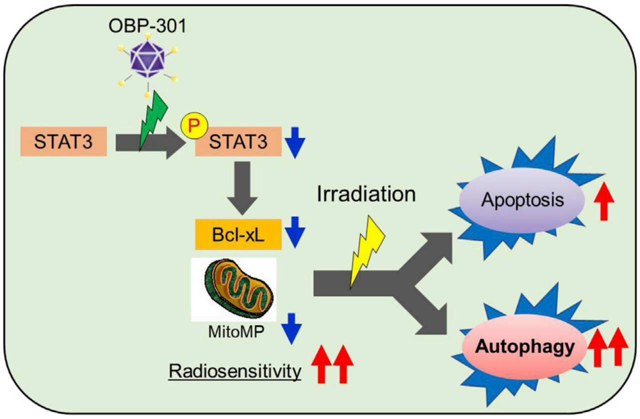

Figure 7. Schematic representation of the mechanism through which OBP-301 regulates radiation-induced apoptosis and autophagy through STAT3 in OSCC

OBP-301 leads to suppression of Bcl-xL expression via suppression of STAT3 phosphorylation. As a result, radiation-induced cell death via apoptosis and autophagy is promoted, and radiosensitivity of OSCC cells is enhanced. Bcl-xL, B cell lymphoma extra large; OSCC, oral squamous cell carcinoma; p-STAT3, phosphorylated signal transducer and activator of transcription 3; PDX, patient-derived xenograft; IR, irradiation; vp, viral particles.

AVAILABILITY OF DATA AND MATERIALS

The datasets generated and/or analyzed in this study are not publicly available, but they are available from the corresponding author on reasonable request.

SUPPLEMENTAL INFORMATION

Supplemental information can be found online at https://doi.org/10.1016/j.omto.2022.10.001.

ACKNOWLEDGMENTS

The authors would like to thank Enago (www.enago.jp) for the English language review. This study was supported by a Grant-in-Aid for Scientific Research (C) (grant 18K09771) from the Japanese Ministry of Education, Culture, Sports, Science, and Technology.

AUTHOR CONTRIBUTIONS

Conceptualization, S.G., Y.U., and R.Y.; experimentation, K.S., S.G., K.Y., R.K., H. Nakashima, H.A., S.K., Y.N., A.I., H.T., and M.H.; investigation, J.I., R.K., J.S., R.Y., K.K., M.N., and A.H.; data curation, Y.M., R.Y., K.K, M.N., A.H., and Y.K.; writing – original draft, S.G. and R.Y.; writing – review & editing, H.T., T.F., and H. Nakayama; supervision, Y.K., M.F., and H. Nakashima; project administration, R.Y. and H. Nakayama; funding acquisition, Y.M., R.Y. and H. Nakayama. All authors have read and agreed to the published version of the manuscript.

DECLARATION OF INTERESTS

The authors declare no competing interests.

REFERENCES

1. Siegel, R.L., Miller, K.D., Fuchs, H.E., and Jemal, A. (2021). Cancer statistics, 2021. CA Cancer J. Clin. *71*, 7–33.
2. Barnes, L., Eveson, J.W., Sidransky, D., and Reichart, P. (2015). Pathology and Genetics of Head and Neck Tumours (IARC).
3. Hanahan, D., and Weinberg, R.A. (2011). Hallmarks of cancer: the next generation. Cell *144*, 646–674.
4. Yamamoto, V.N., Thylur, D.S., Bauschard, M., Schmale, I., and Sinha, U.K. (2016). Overcoming radioresistance in head and neck squamous cell carcinoma. Oral Oncol. *63*, 44–51.
5. Kuwahara, Y., Mori, M., Oikawa, T., Shimura, T., Ohtake, Y., Mori, S., Ohkubo, Y., and Fukumoto, M. (2010). The modified high-density survival assay is the useful tool to predict the effectiveness of fractionated radiation exposure. J. Radiat. Res. *51*, 297–302.
6. Fukumoto, M. (2017). Clinically relevant radioresistant cell line: a simple model to understand cancer radioresistance. Med. Mol. Morphol. *50*, 195–204.
7. Hori, M., Matsuda, T., Shibata, A., Katanoda, K., Sobue, T., and Nishimoto, H.; Japan Cancer Surveillance Research Group (2015). Cancer incidence and incidence rates in Japan in 2009: a study of 32 population-based cancer registries for the Monitoring of Cancer Incidence in Japan (MCIJ) project. Jpn. J. Clin. Oncol. *45*, 884–891.
8. NCCN Clinical Practice Guidelines; Older Adult Oncology. http://www.nccn.org/professionals/physician_gls/f_guidelines.asp.
9. Kawashima, T., Kagawa, S., Kobayashi, N., Shirakiya, Y., Umeoka, T., Teraishi, F., Taki, M., Kyo, S., Tanaka, N., and Fujiwara, T. (2004). Telomerase-specific replication-selective virotherapy for human cancer. Clin. Cancer Res. *10*, 285–292.
10. Li, D., Duan, L., Freimuth, P., and O'Malley, B.W., Jr. (1999). Variability of adenovirus receptor density influences gene transfer efficiency and therapeutic response in head and neck cancer. Clin. Cancer Res. *5*, 4175–4181.
11. Nakayama, J., Tahara, H., Tahara, E., Saito, M., Ito, K., Nakamura, H., Nakanishi, T., Tahara, E., Ide, T., and Ishikawa, F. (1998). Telomerase activation by hTRT in human normal fibroblasts and hepatocellular carcinomas. Nat. Genet. *18*, 65–68.
12. Hashimoto, Y., Watanabe, Y., Shirakiya, Y., Uno, F., Kagawa, S., Kawamura, H., Nagai, K., Tanaka, N., Kumon, H., Urata, Y., and Fujiwara, T. (2008). Establishment of biological and pharmacokinetic assays of telomerase-specific replication-selective adenovirus. Cancer Sci. *99*, 385–390.
13. Liu, D., Kojima, T., Ouchi, M., Kuroda, S., Watanabe, Y., Hashimoto, Y., Onimatsu, H., Urata, Y., and Fujiwara, T. (2009). Preclinical evaluation of synergistic effect of telomerase-specific oncolytic virotherapy and gemcitabine for human lung cancer. Mol. Cancer Ther. *8*, 980–987.
14. Tanimoto, T., Tazawa, H., Ieda, T., Nouso, H., Tani, M., Oyama, T., Urata, Y., Kagawa, S., Noda, T., and Fujiwara, T. (2020). Elimination of MYCN-amplified neuroblastoma cells by telomerase-targeted oncolytic virus via MYCN suppression. Mol. Ther. Oncol. *18*, 14–23.
15. Nemunaitis, J., Tong, A.W., Nemunaitis, M., Senzer, N., Phadke, A.P., Bedell, C., Adams, N., Zhang, Y.A., Maples, P.B., Chen, S., et al. (2010). A phase I study of telomerase-specific replication competent oncolytic adenovirus (telomelysin) for various solid tumors. Mol. Ther. *18*, 429–434.
16. Shirakawa, Y., Tazawa, H., Tanabe, S., Kanaya, N., Noma, K., Koujima, T., Kashima, H., Kato, T., Kuroda, S., Kikuchi, S., et al. (2021). Phase I dose-escalation study of endoscopic intratumoral injection of OBP-301 (Telomelysin) with radiotherapy in oesophageal cancer patients unfit for standard treatments. Eur. J. Cancer *153*, 98–108.
17. Fujita, K., Kimura, M., Kondo, N., Sakakibara, A., Sano, D., Ishiguro, Y., and Tsukuda, M. (2008). Anti-tumor effects of telomelysin for head and neck squamous cell carcinoma. Oncol. Rep. *20*, 1363–1368.
18. Kurihara, Y., Watanabe, Y., Onimatsu, H., Kojima, T., Shirota, T., Hatori, M., Liu, D., Kyo, S., Mizuguchi, H., Urata, Y., et al. (2009). Telomerase-specific virotheranostics for human head and neck cancer. Clin. Cancer Res. *15*, 2335–2343.
19. Sakakibara, A., Tsukuda, M., Kondo, N., Ishiguro, Y., Kimura, M., Fujita, K., Takahashi, H., and Matsuda, H. (2011). Examination of the optimal condition on

the in vitro sensitivity to telomelysin in head and neck cancer cell lines. Auris Nasus Larynx *38*, 589–599.

20. Kondo, N., Tsukuda, M., Kimura, M., Fujita, K., Sakakibara, A., Takahashi, H., Ishiguro, Y., Toth, G., and Matsuda, H. (2010). Antitumor effects of telomelysin in combination with paclitaxel or cisplatin on head and neck squamous cell carcinoma. Oncol. Rep. *23*, 355–363.
21. Kuroda, S., Fujiwara, T., Shirakawa, Y., Yamasaki, Y., Yano, S., Uno, F., Tazawa, H., Hashimoto, Y., Watanabe, Y., Noma, K., et al. (2010). Telomerase-dependent oncolytic adenovirus sensitizes human cancer cells to ionizing radiation via inhibition of DNA repair machinery. Cancer Res. *70*, 9339–9348.
22. Takahashi, H., Hyakusoku, H., Horii, C., Takahashi, M., Nishimura, G., Taguchi, T., Kondo, N., Sakakibara, A., Urata, Y., and Sano, D. (2014). Telomerase-specific oncolytic adenovirus: antitumor effects on radiation-resistant head and neck squamous cell carcinoma cells. Head Neck *36*, 411–418.
23. Baskar, R., Lee, K.A., Yeo, R., and Yeoh, K.W. (2012). Cancer and radiation therapy: current advances and future directions. Int. J. Med. Sci. *9*, 193–199.
24. Kuwahara, Y., Tomita, K., Urushihara, Y., Sato, T., Kurimasa, A., and Fukumoto, M. (2018). Association between radiation-induced cell death and clinically relevant radioresistance. Histochem. Cell Biol. *150*, 649–659.
25. Omori, T., Tazawa, H., Yamakawa, Y., Osaki, S., Hasei, J., Sugiu, K., Komatsubara, T., Fujiwara, T., Yoshida, A., Kunisada, T., et al. (2021). Oncolytic virotherapy promotes radiosensitivity in soft tissue sarcoma by suppressing anti-apoptotic MCL1 expression. PLOS ONE *16*, e0250643.
26. Kanaya, N., Kuroda, S., Kakiuchi, Y., Kumon, K., Tsumura, T., Hashimoto, M., Morihiro, T., Kubota, T., Aoyama, K., Kikuchi, S., et al. (2020). Immune modulation by telomerase-specific oncolytic adenovirus synergistically enhances antitumor efficacy with anti-PD1 antibody. Mol. Ther. *28*, 794–804.
27. Lee, S.Y., Park, H.R., Cho, N.H., Choi, Y.P., Rha, S.Y., Park, S.W., and Kim, S.H. (2013). Identifying genes related to radiation resistance in oral squamous cell carcinoma cell lines. Int. J. Oral Maxillofac. Surg. *42*, 169–176.
28. Ishigami, T., Uzawa, K., Higo, M., Nomura, H., Saito, K., Kato, Y., Nakashima, D., Shiiba, M., Bukawa, H., Yokoe, H., et al. (2007). Genes and molecular pathways related to radioresistance of oral squamous cell carcinoma cells. Int. J. Cancer *120*, 2262–2270.
29. M.W, Y., Lin, H.Y., Chiou, W.Y., Lin, R.I., Chen, C.A., Lee, M.S., Chi, C.L., Chen, L.C., Huang, L.W., et al. (2021). IRAK2, an IL1R/TLR immune mediator, enhances radiosensitivity via modulating caspase 8/3-mediated apoptosis in oral squamous cell carcinoma. Front. Oncol. *11*, 647175.
30. Fujiwara, T., Urata, Y., and Tanaka, N. (2007). Telomerase-specific oncolytic virotherapy for human cancer with the hTERT promoter. Curr. Cancer Drug Targets *7*, 191–201.
31. Pankiv, S., Clausen, T.H., Lamark, T., Brech, A., Bruun, J.A., Outzen, H., Øvervatn, A., Bjørkøy, G., and Johansen, T. (2007). p62/SQSTM1 binds directly to Atg8/LC3 to facilitate degradation of ubiquitinated protein aggregates by autophagy. J. Biol. Chem. *282*, 24131–24145.
32. Kim, N.W., Piatyszek, M.A., Prowse, K.R., Harley, C.B., West, M.D., Ho, P.L., Coviello, G.M., Wright, W.E., Weinrich, S.L., and Shay, J.W. (1994). Specific association of human telomerase activity with immortal cells and cancer. Science *266*, 2011–2015.
33. Jie, M.M., Chang, X., Zeng, S., Liu, C., Liao, G.B., Wu, Y.R., Liu, C.H., Hu, C.J., Yang, S.M., and Li, X.Z. (2019). Diverse regulatory manners of human telomerase reverse transcriptase. Cell Commun. Signal. *17*, 63.
34. Sasaki, T., Tazawa, H., Hasei, J., Kunisada, T., Yoshida, A., Hashimoto, Y., Yano, S., Yoshida, R., Uno, F., Kagawa, S., et al. (2011). Preclinical evaluation of telomerase-specific oncolytic virotherapy for human bone and soft tissue sarcomas. Clin. Cancer Res. *17*, 1828–1838.
35. Yamasaki, Y., Tazawa, H., Hashimoto, Y., Kojima, T., Kuroda, S., Yano, S., Yoshida, R., Uno, F., Mizuguchi, H., Ohtsuru, A., et al. (2012). A novel apoptotic mechanism of genetically engineered adenovirus-mediated tumour-specific p53 overexpression through E1A-dependent p21 and MDM2 suppression. Eur. J. Cancer *48*, 2282–2291.
36. Yano, S., Tazawa, H., Hashimoto, Y., Shirakawa, Y., Kuroda, S., Nishizaki, M., Kishimoto, H., Uno, F., Nagasaka, T., Urata, Y., et al. (2013). A genetically engineered oncolytic adenovirus decoys and lethally traps quiescent cancer stem-like cells in S/G2/M phases. Clin. Cancer Res. *19*, 6495–6505.
37. Lin, W.H., Yeh, S.H., Yang, W.J., Yeh, K.H., Fujiwara, T., Nii, A., Chang, S.S., and Chen, P.J. (2013). Telomerase-specific oncolytic adenoviral therapy for orthotopic hepatocellular carcinoma in HBx transgenic mice. Int. J. Cancer *132*, 1451–1462.
38. Kuroda, S., Kikuchi, S., Yoshida, R., Umeda, Y., et al. (2020). Oncolytic virus-mediated targeting of the ERK signaling pathway inhibits invasive propensity in human pancreatic cancer. Mol. Ther. Oncolytics *17*, 107–117.
39. Nakajima, O., Matsunaga, A., Ichimaru, D., Urata, Y., Fujiwara, T., and Kawakami, K. (2009). Telomerase-specific virotherapy in an animal model of human head and neck cancer. Mol. Cancer Ther. *8*, 171–177.
40. Takakura, M., Nakamura, M., Kyo, S., Hashimoto, M., Mori, N., Ikoma, T., Mizumoto, Y., Fujiwara, T., Urata, Y., and Inoue, M. (2010). Intraperitoneal administration of telomerase-specific oncolytic adenovirus sensitizes ovarian cancer cells to cisplatin and affects survival in a xenograft model with peritoneal dissemination. Cancer Gene Ther. *17*, 11–19.
41. Ishikawa, W., Kikuchi, S., Ogawa, T., Tabuchi, M., Tazawa, H., Kuroda, S., Noma, K., Nishizaki, M., Kagawa, S., Urara, Y., et al. (2020). Boosting replication and penetration of oncolytic adenovirus by paclitaxel eradicate peritoneal metastasis of gastric cancer mol. Ther. Oncol. *18*, 262–271.
42. You, L., Wang, Z., Li, H., Shou, J., Jing, Z., Xie, J., Sui, X., Pan, H., and Han, W. (2015). The role of STAT3 in autophagy. Autophagy *11*, 729–739.
43. Wang, X., Zhang, X., Qiu, C., and Yang, N. (2020). STAT3 contributes to radioresistance in cancer. Front. Oncol. *10*, 1120.
44. Farmer, T., O'Neill, K.L., Naslavsky, N., Luo, X., and Caplan, S. (2019). Retromer facilitates the localization of Bcl-xL to the mitochondrial outer membrane. Mol. Biol. Cell *30*, 1138–1146.
45. Li, M., Wang, D., He, J., Chen, L., and Li, H. (2020). Bcl-XL: a multifunctional anti-apoptotic protein. Pharmacol. Res. *151*, 104547.
46. Kim, S.Y., Song, X., Zhang, L., Bartlett, D.L., and Lee, Y.J. (2014). Role of Bcl-xL/Beclin-1 in interplay between apoptosis and autophagy in oxaliplatin and bortezomib-induced cell death. Biochem. Pharmacol. *88*, 178–188.
47. Cekanova, M., and Rathore, K. (2014). Animal models and therapeutic molecular targets of cancer: utility and limitations. Drug Des. Devel. Ther. *8*, 1911–1921.
48. Huang, P., Kaku, H., Chen, J., Kashiwakura, Y., Saika, T., Nasu, Y., Urata, Y., Fujiwara, T., Watanabe, M., and Kumon, H. (2010). Potent antitumor effects of combined therapy with a telomerase-specific, replication-competent adenovirus (OBP-301) and IL-2 in a mouse model of renal cell carcinoma. Cancer Gene Ther. *17*, 484–491.
49. Siolas, D., and Hannon, G.J. (2013). Patient-derived tumor xenografts: transforming clinical samples into mouse models. Cancer Res. *73*, 5315–5319.
50. Adelstein, D.J., Li, Y., Adams, G.L., Wagner, H., Jr., Kish, J.A., Ensley, J.F., Schuller, D.E., and Forastiere, A.A. (2003). An intergroup phase III comparison of standard radiation therapy and two schedules of concurrent chemoradiotherapy in patients with unresectable squamous cell head and neck cancer. J. Clin. Oncol. *21*, 92–98.
51. Bonner, J.A., Harari, P.M., Giralt, J., Azarnia, N., Shin, D.M., Cohen, R.B., Jones, C.U., Sur, R., Raben, D., Jassem, J., et al. (2006). Radiotherapy plus cetuximab for squamous-cell carcinoma of the head and neck. N. Engl. J. Med. *354*, 567–578.
52. Ghosh, S. (2019). Cisplatin: the first metal based anticancer drug. Bioorg. Chem. *88*, 102925.
53. Cetuximab hypersensitivity infusion reactions: incidence and risk factors. J. Oncol. Pharm. Pract. 19, 222–227.
54. Ishisaki, A., Oida, S., Momose, F., Amagasa, T., Rikimaru, K., Ichijo, H., and Sasaki, S. (1994). Identification and characterization of autocrine-motility-factor-like activity in oral squamous-cell-carcinoma cells. Int. J. Cancer *59*, 783–788.
55. Hayashi, E., Rikimaru, K., and Nagayama, M. (1995). Simultaneous production of G- and M-CSF by an oral cancer cell line and the synergistic effects on associated leucocytosis. Eur. J. Cancer B Oral Oncol. *31B*, 323–327.

56. Chou, T.C. (2006). Theoretical basis, experimental design, and computerized simulation of synergism and antagonism in drug combination studies. Pharmacol. Rev. *58*, 621–681.

57. Nomura, T., Murakami, R., Toya, R., Teshima, K., Nakahara, A., Hirai, T., Hiraki, A., Nakayama, H., Yoshitake, Y., Ota, K., et al. (2010). Phase II study of preoperative concurrent chemoradiation therapy with S-1 in patients with T4 oral squamous cell carcinoma. Int. J. Radiat. Oncol. Biol. Phys. *76*, 1347–1352.

58. Edge, S., Byrd, D.R., and Compton, C. (2010). AJCC Cancer Staging Manual (Springer).

59. Shimosato, Y., Oboshi, S., and Baba, K. (1971). Histological evaluation of effects of radiotherapy and chemotherapy for carcinomas. Jpn. J. Clin. Oncol. *1*, 19–35.

60. Allred, D.C., Bustamante, M.A., Daniel, C.O., Gaskill, H.V., and Cruz, A.B., Jr. (1990). Immunocytochemical analysis of estrogen receptors in human breast carcinomas. Evaluation of 130 cases and review of the literature regarding concordance with biochemical assay and clinical relevance. Arch. Surg. *125*, 107–113.

OMTO, Volume *27*

Supplemental information

An oncolytic virus as a promising candidate for the treatment of radioresistant oral squamous cell carcinoma

Shunsuke Gohara, Kosuke Shinohara, Ryoji Yoshida, Ryusho Kariya, Hiroshi Tazawa, Masashi Hashimoto, Junki Inoue, Ryuta Kubo, Hikaru Nakashima, Hidetaka Arita, Sho Kawaguchi, Keisuke Yamana, Yuka Nagao, Asuka Iwamoto, Junki Sakata, Yuichiro Matsuoka, Hisashi Takeshita, Masatoshi Hirayama, Kenta Kawahara, Masashi Nagata, Akiyuki Hirosue, Yoshikazu Kuwahara, Manabu Fukumoto, Seiji Okada, Yasuo Urata, Toshiyoshi Fujiwara, and Hideki Nakayama

Supplementary Materials and Methods

Immunohistochemical staining analysis

Immunohistochemical staining analysis was performed basically as described in main manuscript. We semi-quantified the proportion of Adenovirus-2/5 E1A (E1A)-positive cells among the total number of cancer cells and the staining intensity for E1A. The proportion score of E1A-positive cells was classified as follows: 0, <1%; 1, 1%–10%; 2, 11%–50%; and 3, >50%. The intensity score was classified as follows: 0, lack of intensity; 1, weak, detectable only in high-power fields; 2, moderate, detectable in low-power fields; and 3, strong. The proportion and intensity scores were summed to produce a E1A score ranging 0–6. The HMGB1 and Calreticulin expression was determined by calculating the number of positive cells out of 100 in five random fields (400 × objective). A percentage of positive cells was calculated by dividing the number of positive cells by the total number of cells per sample and multiplying by 100. The antibodies used in above analyses were shown in Supplementary Table S3. In the immunohistochemical staining of tumor tissues obtained from PDX model, the appropriate antibodies shown in Supplementary Table S3 were used.

Histological assessment of vital organs in mice

To evaluate the adverse effects of OBP-301 on vital organs, the heart, liver, and lungs were removed at the end of the treatment experiment. The removed organs were fixed in 10% buffered formalin and H&E stained for histological evaluation.

RT-PCR

Total RNA s isolated using the FastGeneTM RNA Basic Kit (NIPPON Genetics, Tokyo, Japan) and reverse transcribed into cDNA using the ReverTra Ace qPCR RT Kit (Toyobo, Osaka, Japan). PCR was performed using the Thunderbird SYBR qPCR Mix (Toyobo). Data obtained from RT-qPCR were analyzed using the 2−ΔΔCt method, with *glyceraldehyde-3-phosphate dehydrogenase (GAPDH)* as a housekeeping gene. Each sample was run in triplicate. The following primers were used: *E1A* (forward, 5-CCTGAGACGCCCGACATC-3; reverse, 5-GGACCGGAGTCACAGCTATCC-3); *GAPDH* (forward, 5-CAACAGCCTCAAGATCATCAGC-3; reverse, 5-TTCTAGACGGCAGGTCAGGTC-3).

Supplementary Table S1. Antibdy lists

Antigens	Species Antibodies Raised in	Dilution	Supplier, Catalog No.
hTERT	Mouse, monoclonal	1/1000	MBL, #M216-3
CAR	Rabbit, monoclonal	1/1000	Cell Signaling Technology, #16984
STAT3	Rabbit, monoclonal	1/1000	Cell Signaling Technology, #4904
p-STAT3	Rabbit, monoclonal	1/1000	Cell Signaling Technology, #9145
Bcl-xL	Rabbit, monoclonal	1/1000	Cell Signaling Technology, #2764
Cleaved caspase3	Rabbit, monoclonal	1/1000	Cell Signaling Technology, #9664
LC3 I/II	Rabbit, monoclonal	1/1000	Cell Signaling Technology, #12741
p62	Rabbit, polyclonal	1/1000	MBL, #PM045
β-actin	Mouse, monoclonal	1/2000	Sigma-Aldrich, A5316

Supplementary Table S2. Primer sets for RT-qPCR

Gene	sequence 5'-3' Forward	sequence 5'-3' Reverse
hTERT	TTCCTGCACTGGCTGATGAGTGT	CGCTCGGCCCTCTTTTCTCTG
CAR	AGCCTTCAGGTGCGAGATGTTACG	TACGACAGCAAAAGATGATAAGAC
GAPDH	GGGAAGGTGAAGGTCGGAGTC	AGCAGAGGGGGCAGAGATGAT

Supplementary Table S3. Antibdy lists

Antigens	Species Antibodies Raised in	Dilution	Supplier, Catalog No.
hTERT	Mouse, monoclonal	1/100	Wako, #TMab-6
p-STAT3	Rabbit, monoclonal	1/100	Cell Signaling Technology, #9145
Bcl-xL	Rabbit, monoclonal	1/200	Cell Signaling Technology, #2764
Anti-Coxsackie Adenovirus Receptor/hCAR	Rabbit, polyclonal	1/100	Abcam, ab133380
Adenovirus-2/5 E1A	Rabbit, polyclonal	1/100	Santa Cruz Biothechnology, M73
p62	Rabbit, polyclonal	1/200	Proteintech, 18420-0-AP
HMGB1	Rabbit, polyclonal	1/100	Proteintech, 10829-1-AP
Calreticulin	Rabbit, polyclonal	1/200	Proteintech, 27298-1-AP

A

RT-PCR

CAR

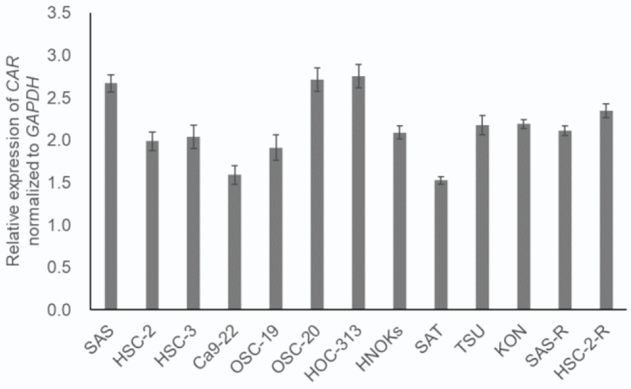

hTERT

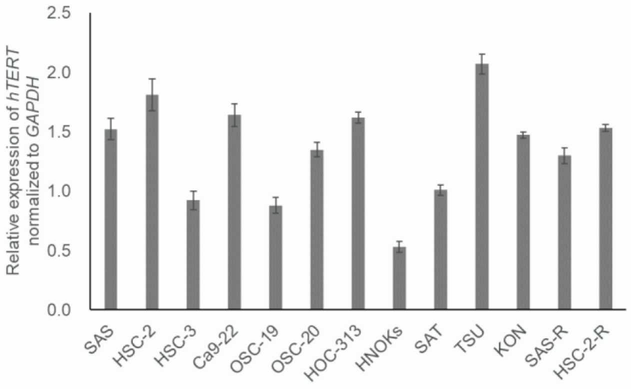

B

Western blotting

CAR

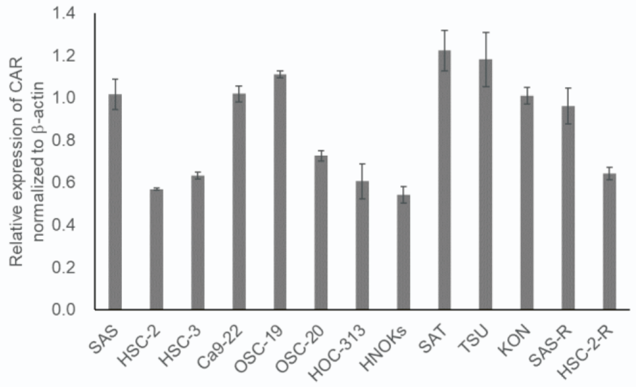

hTERT

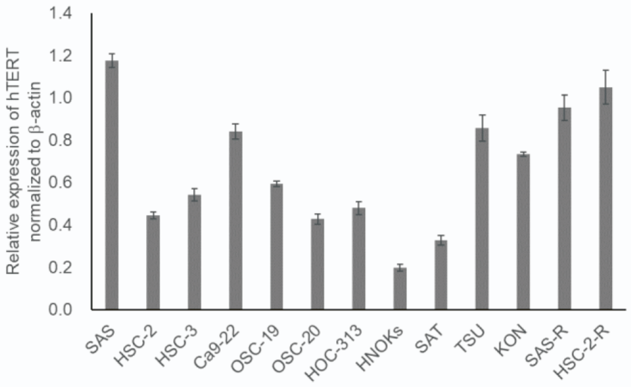

Figure S1. Results of semi-quantitative analysis of RT-PCR and western blotting.

A) The graph of semi-quantitative analysis of RT-PCR.. The result of CAR (lest panel). The result of hTERT (right panel). The results are shown as the means ± s.d. of three independent experiments.

B) The graph of semi-quantitative analysis of western blotting. These results are shown as the means of at least three independent experiments performed in triplicate. The result of CAR (lest panel). The result of hTERT (right panel). The results are shown as the means ± s.d. of three independent experiments.

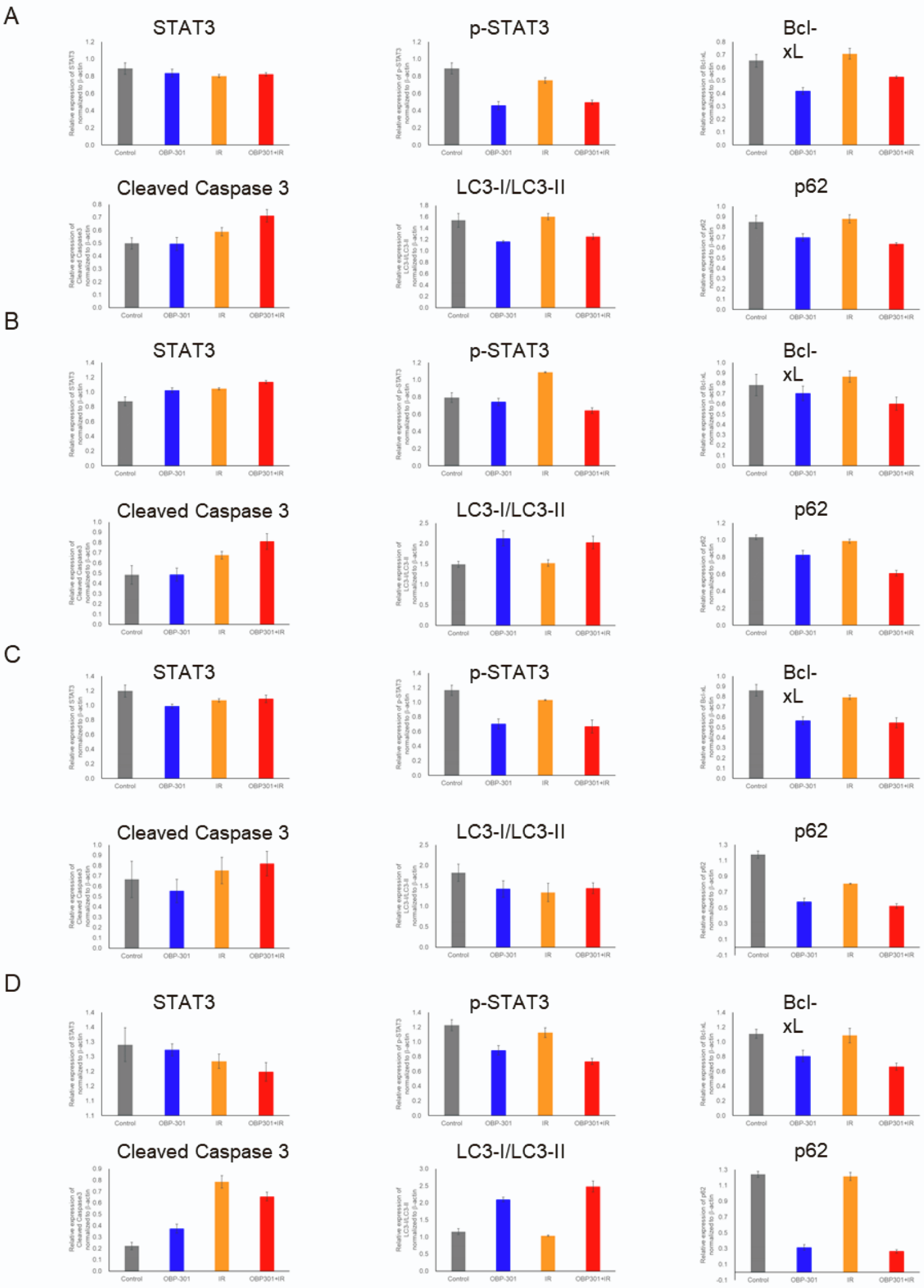

Figure S2. Results of semi-quantitative analysis of western blotting.

A) The graph of semi-quantitative analysis of western blotting in SAS. The results are shown as the means ± s.d. of three independent experiments.

B) The graph of semi-quantitative analysis of western blotting in SAS-R. The results are shown as the means ± s.d. of three independent experiments.

C) The graph of semi-quantitative analysis of western blotting in HSC-2. The results are shown as the means ± s.d. of three independent experiments.

D) The graph of semi-quantitative analysis of western blotting in HSC-2-R. The results are shown as the means ± s.d. of three independent experiments.

A

Age	83
Sex	Female
Primary site	Right of the tongue
cTNM	cT3N0M0
Pattern of invasion	WPOI-5
Initial Treatment	Extensive surgery
Prognosis	Dead from cancer

B

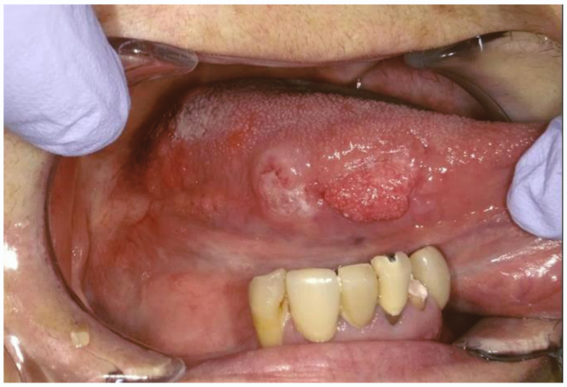

C

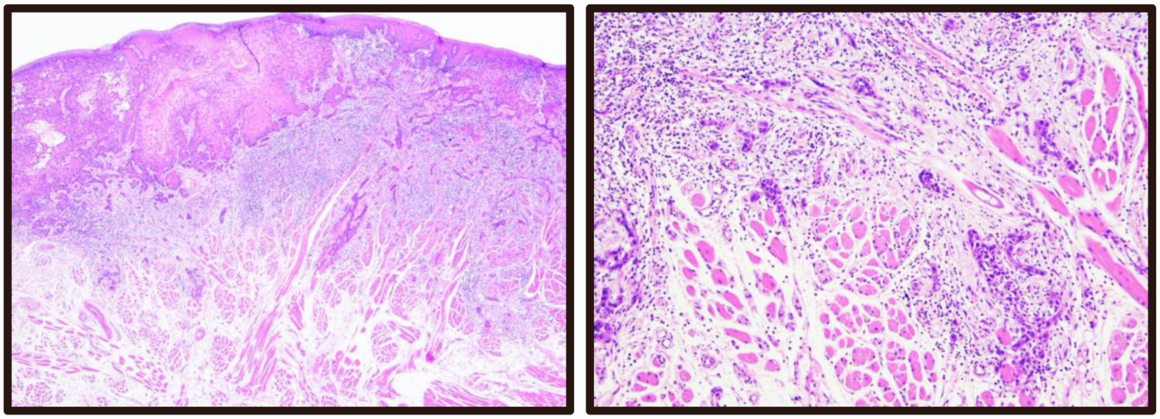

D

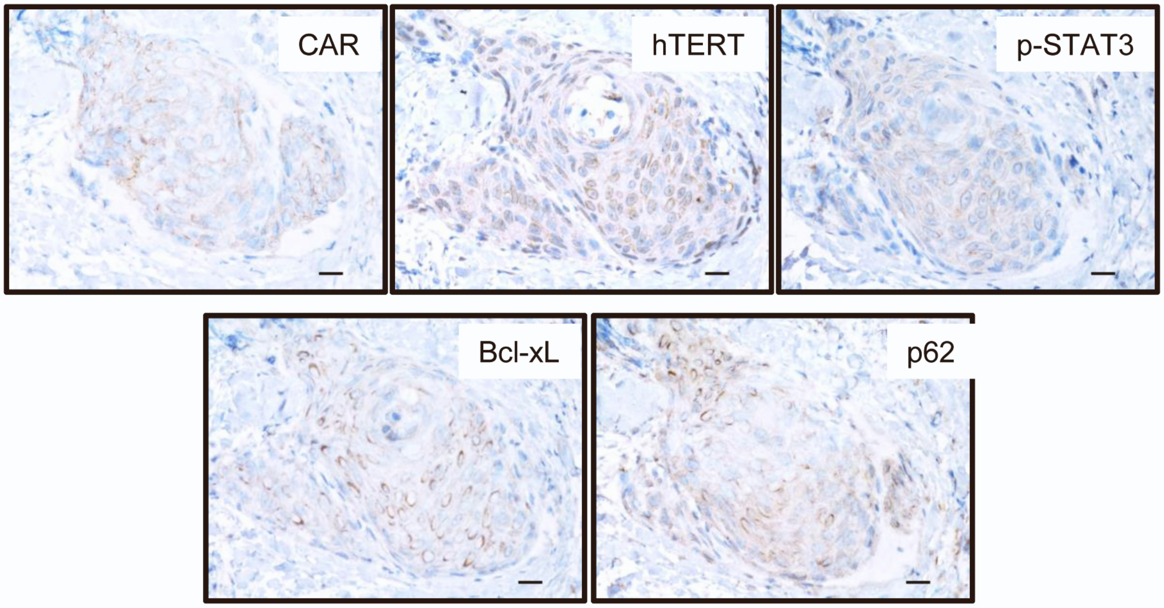

Figure S3. Patient information used to establish the PDX model.

A) Detailed clinical information of the patient.
B) Intraoral photographs of the patient.
C) Representative hematoxylin and eosin staining photomicrographs of the resected tumor (Right; High-power field, Left; Low-power field).
D) The results of immunohistochemical staining of tumors in PDX model under no treatment condition.

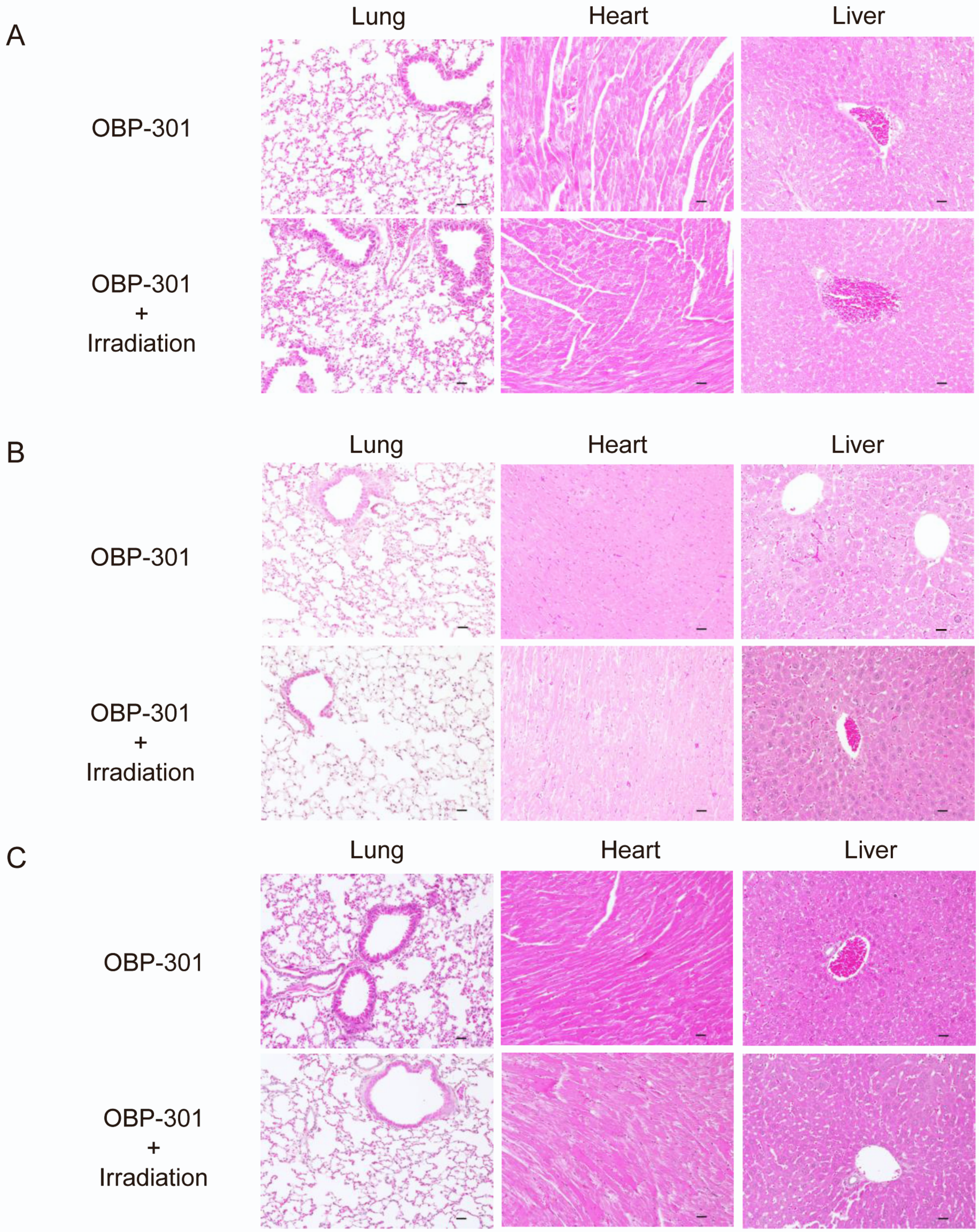

Figure S4. Histology of vital organs in a mouse subcutaneous transplantation model with treatment experiments.

A) Representative hematoxylin and eosin staining of vital organs in a mouse model of subcutaneously implanted SAS. Original magnification, ×200. Scale bar: 20 μm.

B) Representative hematoxylin and eosin staining of vital organs in a mouse model of subcutaneously implanted SAS-R. Original magnification, ×200. Scale bar: 20 μm.

C) Representative hematoxylin and eosin staining of vital organs in a PDX model. Original magnification, ×200. Scale bar: 20 μm.

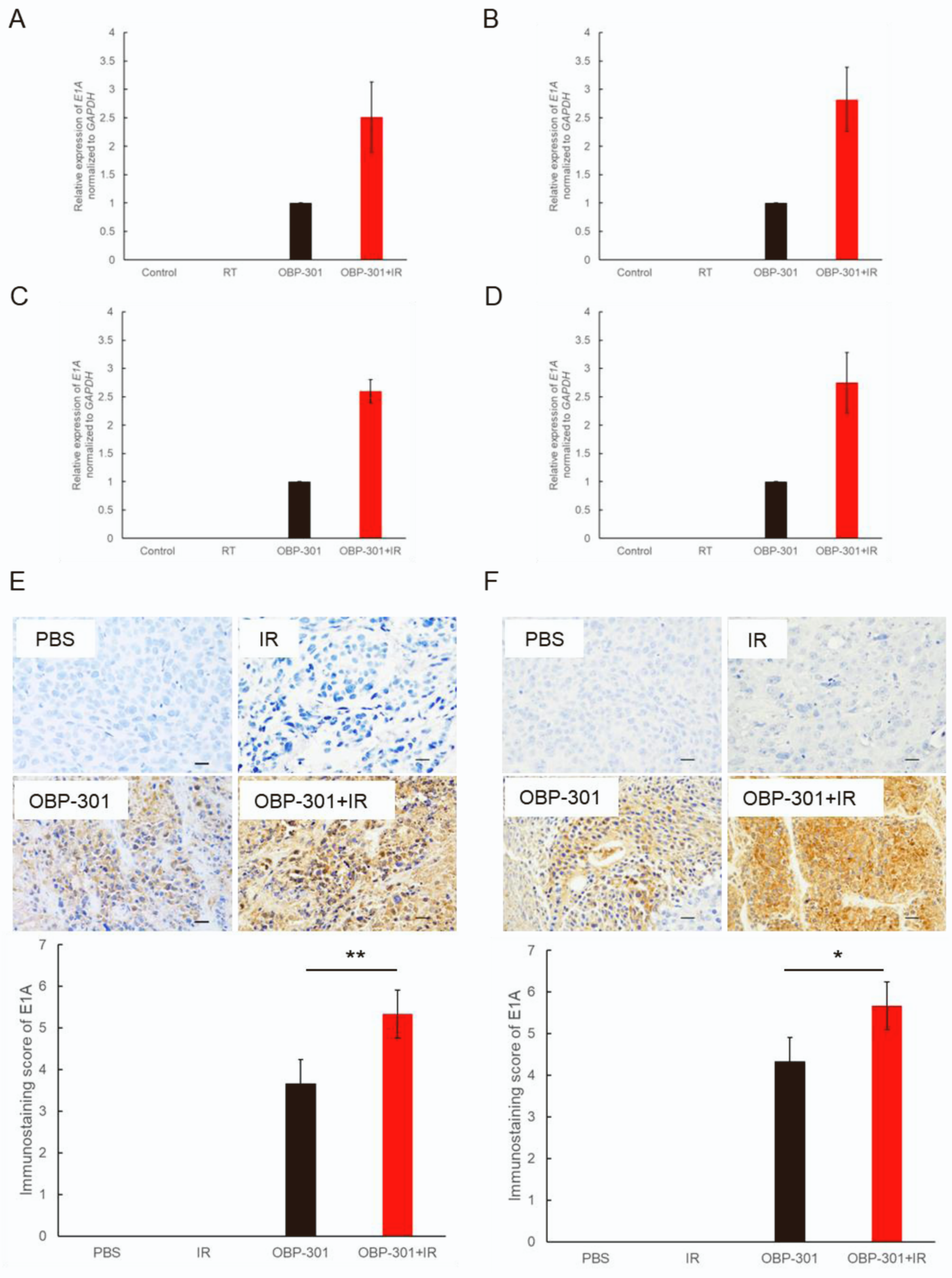

Figure S5. Analysis of the effect of irradiation on the infection efficiency of OBP-301.
Representative immunohistochemical stainings of E1A in cell-derived xenograft model by using SAS (upper). NT, no treatment; RT, radiotherapy. Original magnification, ×400. Scale bar=20 mm. The immunostaining score obtained from three independent experiments were calculated and statistically analyzed. The results are shown as the means ± s.d. of three independent experiments (bottom). *$p < 0.05$.
Representative immunohistochemical stainings of E1A in cell-derived xenograft model by using SAS-R (upper). NT, no treatment; RT, radiotherapy. Original magnification, ×400. Scale bar =20 mm. The immunostaining score obtained from three independent experiments were calculated and statistically analyzed. The results are shown as the means ± s.d. of three independent experiments (bottom). **$p < 0.01$.

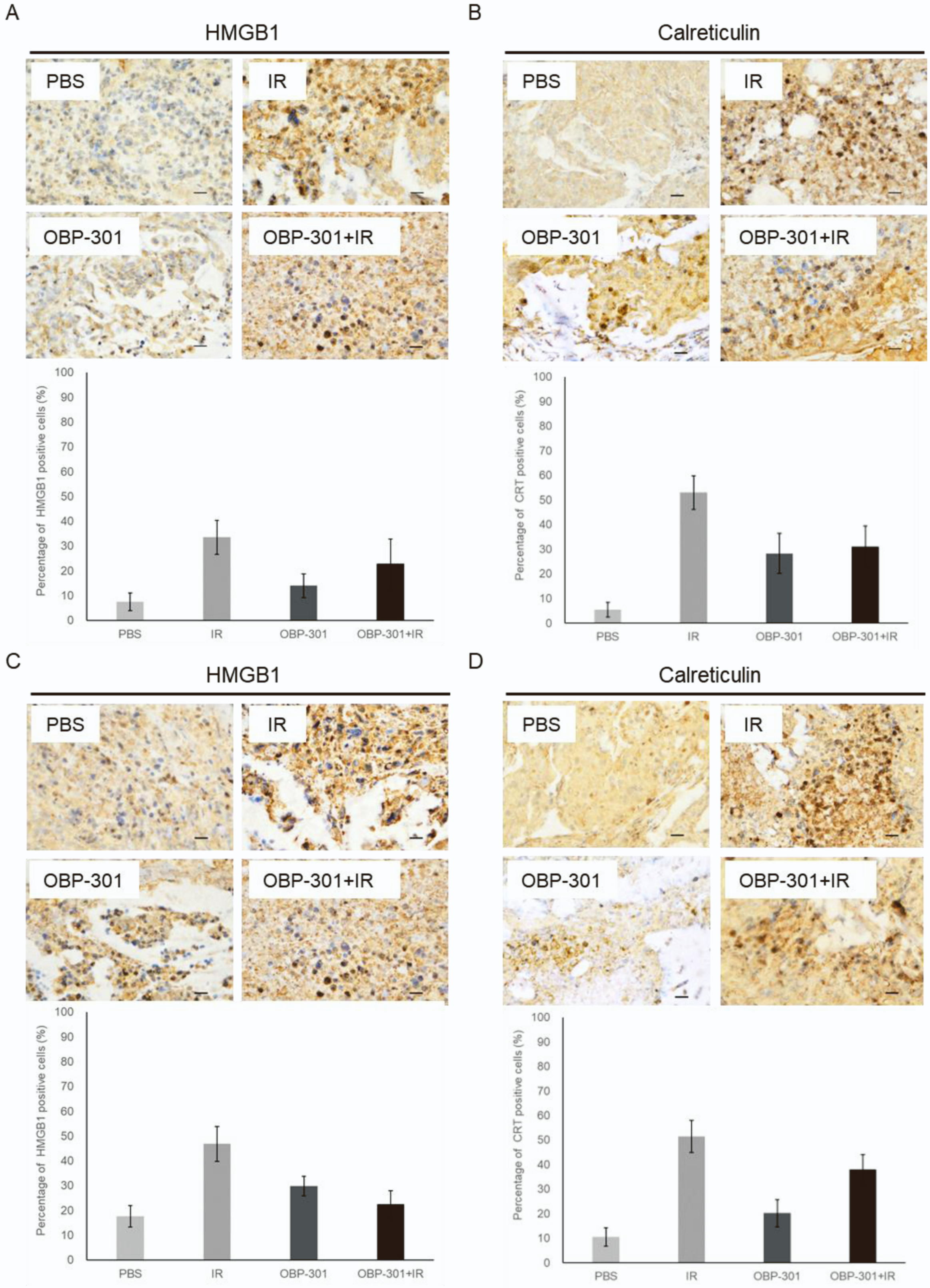

Figure S6. Immunohistochemical staining analysis of HMGB1 and calreticulin *in vivo.*
(A-D) Representative images of immunostaining of HMGB1 and Calreticulin using samples obtained from the CDX model after the experiment (upper). Scale bar: 20 μm. The number of positive cells obtained from three independent experiments were calculated and statistically analyzed. The results are shown as the means ± s.d. of three independent experiments (bottom).

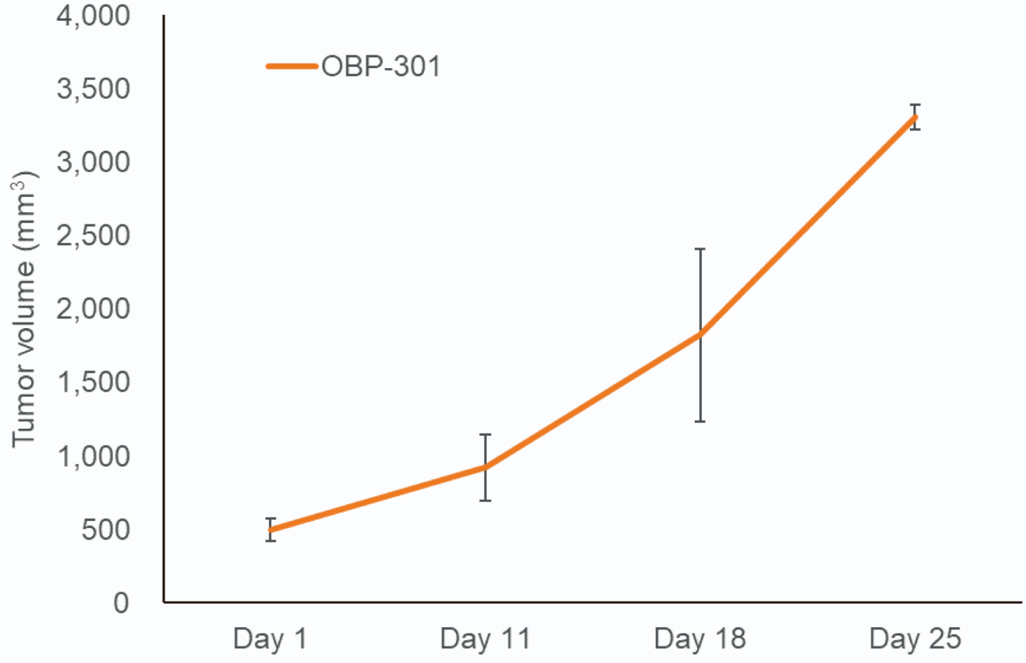

Figure S7. Preliminary experiments with OBP-301 monotherapy in PDX model.
The graphs of tumor volume transition of OBP-301 monotherapy in PDX model. Mean ± S.D. of three independent experiments was calculated. n=3 per group.
